# Integrative Approach for Precise Genotyping and Transcriptomics of Salt Tolerant Introgression Rice Lines

**DOI:** 10.3389/fpls.2021.797141

**Published:** 2022-01-21

**Authors:** Mireia Bundó, Héctor Martín-Cardoso, Michele Pesenti, Jorge Gómez-Ariza, Laia Castillo, Julien Frouin, Xavier Serrat, Salvador Nogués, Brigitte Courtois, Cécile Grenier, Gian Attilio Sacchi, Blanca San Segundo

**Affiliations:** ^1^Centre for Research in Agricultural Genomics, CSIC-IRTA-UAB-UB, Bellaterra, Spain; ^2^Department of Agricultural and Environmental Sciences – Production, Landscape, Agroenergy – DiSAA, University of Milan, Milan, Italy; ^3^CIRAD, UMR AGAP, Montpellier, France; ^4^AGAP, CIRAD, INRAE, Institut Agro, University of Montpellier, Montpellier, France; ^5^Departament de Biologia Evolutiva, Ecologia i Ciències Ambientals, Secció de Fisiologia Vegetal, Universitat de Barcelona, Barcelona, Spain; ^6^Consejo Superior de Investigaciones Científicas, Barcelona, Spain

**Keywords:** genotyping by sequencing (GBS), *Oryza sativa* (L.), *Saltol*, transcriptomics, *indica* and *japonica*, introgression lines (ILs), salinity stress

## Abstract

Rice is the most salt sensitive cereal crop and its cultivation is particularly threatened by salt stress, which is currently worsened due to climate change. This study reports the development of salt tolerant introgression lines (ILs) derived from crosses between the salt tolerant *indica* rice variety FL478, which harbors the *Saltol* quantitative trait *loci* (QTL), and the salt-sensitive *japonica* elite cultivar OLESA. Genotyping-by-sequencing (GBS) and Kompetitive allele specific PCR (KASPar) genotyping, in combination with step-wise phenotypic selection in hydroponic culture, were used for the identification of salt-tolerant ILs. Transcriptome-based genotyping allowed the fine mapping of *indica* genetic introgressions in the best performing IL (IL22). A total of 1,595 genes were identified in *indica* regions of IL22, which mainly located in large introgressions at Chromosomes 1 and 3. In addition to *OsHKT1;5*, an important number of genes were identified in the introgressed *indica* segments of IL22 whose expression was confirmed [e.g., genes involved in ion transport, callose synthesis, transcriptional regulation of gene expression, hormone signaling and reactive oxygen species (ROS) accumulation]. These genes might well contribute to salt stress tolerance in IL22 plants. Furthermore, comparative transcript profiling revealed that *indica* introgressions caused important alterations in the background gene expression of IL22 plants (*japonica* cultivar) compared with its salt-sensitive parent, both under non-stress and salt-stress conditions. In response to salt treatment, only 8.6% of the salt-responsive genes were found to be commonly up- or down-regulated in IL22 and OLESA plants, supporting massive transcriptional reprogramming of gene expression caused by *indica* introgressions into the recipient genome. Interactions among *indica* and *japonica* genes might provide novel regulatory networks contributing to salt stress tolerance in introgression rice lines. Collectively, this study illustrates the usefulness of transcriptomics in the characterization of new rice lines obtained in breeding programs in rice.

## Introduction

Rice (*Oryza sativa* L.) is one of the most important cereal crops in the world and serves as a staple food for more than half of the world’s population. Rice is also considered a salt-sensitive crop, particularly at the seedling and reproductive stages (Zeng and Shannon, 2000). The extent and severity of salt-affected agricultural land is predicted to worsen as a result of inadequate drainage of irrigated land, entry of sea water in coastal rice fields, and global warming.

Tolerance to salt stress in plants is a complex trait governed by a multitude of physiological and molecular mechanisms (Van Zelm et al., 2020). High salinity imposes an immediate osmotic stress, manifested by reduced water uptake and growth retardation, followed by a long-lasting ionic stress due to gradual accumulation of Na^+^, and subsequent secondary stresses, such as oxidative stress and nutritional disorders (Negrão et al., 2011; Van Zelm et al., 2020). In order to maintain fairly constant levels of NaCl over time, roots can regulate NaCl levels by exporting it to the soil or to the shoot. Compared with roots, the transcriptional reprogramming of plant leaves during adaptation to salt stress has deserved less attention. Leaves are, however, more sensitive to salt stress than roots.

In shoots, high concentrations of Na^+^ provoke disruption of the ion homeostasis mechanisms which, in turn, causes a range of metabolic problems. Among other effects, Na^+^ competes with K^+^ for essential cellular functions (e.g., Na^+^ replaces K^+^ at binding sites on enzymes resulting on enzyme deactivation) and the chloride ion may interfere with anionic sites involved in binding of RNA and anionic metabolites. Maintenance of low Na^+^/K^+^ ratio in shoots is then essential to circumvent Na^+^ toxicity under salt stress conditions. For this, plants have multiple Na^+^ transport systems aiming Na^+^ extrusion out of cells, compartmentalization into vacuoles, or sequestration of toxic Na^+^ into older leaves (Wang et al., 2018; Ketehouli et al., 2019; Van Zelm et al., 2020). Members of the high-affinity K^+^ transporter (HKT) transporter/channel family are known to play crucial roles in maintenance of Na^+^ homeostasis during salt stress, and also aquaporins which are crucial for water homeostasis and transport of certain solutes during adaptation to salt stress (Afzal et al., 2016; Wang et al., 2018; Ketehouli et al., 2019). Exposure to salinity causes plasma membrane remodeling affecting the activity of membrane proteins (and signaling molecules) that results in alterations in membrane permeability (Guo et al., 2019). Salt stress also induces changes in the cell wall composition that can benefit the plant by preventing water loss and altering ion transport pathways (Zagorchev et al., 2014).

Increases in cytosolic Ca^2+^ concentration and production of reactive oxygen species (ROS) are some of the earliest described salt stress responses (Van Zelm et al., 2020). ROS act as signaling molecules that trigger signal transduction pathways in response to salt stress. However, when in excess, ROS cause irreversible cellular damage due to their capacity to cause oxidative damage to proteins, DNA and lipids. Redox homeostasis in plants is maintained by the activity of antioxidant enzymes (e.g., superoxide dismutases, ascorbate peroxidases, and glutathione-S-transferases), and non-enzymatic systems (e.g., reduced glutathione and flavonoids) (Hossain and Dietz, 2016). Salt stress responses also involve 14-3-3 proteins, and protein phosphorylation processes (Tan et al., 2016; Yang and Guo, 2018; Shah et al., 2021). Salt stress-responsive kinases include calcineurin B-like (CBL)-interacting protein kinases (CIPKs), mitogen-activated protein kinase (MAPK) cascades, and calcium-dependent protein kinases (CPKs), such as the rice OsCPK4, OsCPK12, or OsCPK21 (Asano et al., 2011, 2012; Campo et al., 2014; Shah et al., 2021). Additionally, hormone signaling pathways creates a sophisticated signaling network that results in salt stress tolerance, with abscisic acid (ABA) being considered an important hormone in mediating plant responses to salt stress (Yu et al., 2020). Salt stress-inducible genes that are ABA independent have been described (Yoshida et al., 2014).

Regulation of gene expression during salinity stress is known to be governed by various transcription factor (TF) pathways, including AP2/ERF, bHLH, bZIP, DREB, GATA, HD-Zip, Homeo-box, MADS-box, MYB, NAC, Trihelix, WHIRLY, WOX, WRKY, YABBY, and zinc finger TFs (Hussain et al., 2021; Shah et al., 2021). These TFs might control salinity tolerance through ABA-dependent and ABA-independent signaling pathways, and play essential role in ROS production, chlorophyll content, lipid-peroxidation (Yang and Guo, 2018; Yoon et al., 2020). They regulate downstream genes by binding to cis-regulatory elements in their promoter region. In particular, members of the AP2/ERF TF family have been comprehensively studied in plant responses to salt stress, including DREB (Dehydration-Responsive Element-binding Protein) and ERF (Ethylene response element-binding factors) TFs. DREBs are classified into two subgroups, DREB1 and DREB2 and are induced by salt and dehydration stress in plants (Nakashima et al., 2014). DREB TFs associated with salinity tolerance include *OsDREB1A*, *OsDREBB1C*, *OsDREBB1F*, and *OsDREB2A* (Dubouzet et al., 2003; Wang et al., 2008; Mallikarjuna et al., 2011; Shah et al., 2021). Regarding NAC (NAM, ATAF, and CUC) TFs, their overexpression in transgenic plants has been shown to improve salinity tolerance in many plant species. As an example, transgenic rice plants overexpressing either *OsNAC6* or *OsNAC1* were shown to exhibit strong tolerance to salt stress (Hu et al., 2006; Nakashima et al., 2007). In other studies, *OsZIP23* was found to function as a transcriptional regulator of a wide spectrum of abiotic stress-related genes through an ABA-dependent regulation pathway, and its overexpression in rice confers salt-stress tolerance (Xiang et al., 2008). For additional information on TFs involved in salinity tolerance in plants we refer to recent reviews by Hussain et al. (2021) and Shah et al. (2021). Although there have been many reports describing mechanisms of salinity tolerance in plants, most of these studies have been carried out in the dicotyledonous model plant Arabidopsis. Much remains to learn about how multiple signaling pathways from different cellular compartments are coordinated, and how salt stress responsive genes function during adaptation to salt stress in crop species.

Many efforts are being made by rice breeders to develop high-yielding rice cultivars with improved tolerance to salt stress. A major quantitative trait *loci* (QTL), named *Saltol*, was identified in a recombinant inbred line (RIL) population derived from a cross between the salt tolerant *indica* landrace Pokkali and the salt sensitive *indica* cultivar IR29 (Gregorio et al., 1997; Bonilla et al., 2002). Since then, the *Saltol* QTL has been widely used to introduce seedling stage salt tolerance into modern high yielding rice varieties, through marker-assisted backcrossing (Waziri et al., 2016; Babu et al., 2017; Yadav et al., 2020). The *Saltol* QTL has been mapped on Chromosome 1, and accounts for a low Na^+^/K^+^ ratio in rice shoots under salt stress (Bonilla et al., 2002; Gregorio et al., 2002; Thomson et al., 2010). The *OsHKT1;5* gene located in the *Saltol* region has been proposed to be the responsible gene for the salinity tolerance provided by *Saltol* (Ren et al., 2005; Platten et al., 2013). *OsHKT1;5* is expressed in rice roots and encodes for a Na^+^-selective transporter that mediates Na^+^ exclusion out of the xylem vessel into xylem parenchyma, thus, preventing Na^+^ transfer to young leaf blades (Kobayashi et al., 2017). In this way, *OsHKT1;5* helps in maintaining the Na^+^/K^+^ ratio and minimizes the harmful effects of Na^+^ accumulation in shoots. Although the *Saltol* QTL has been extensively employed in breeding programs to enhance salt tolerance in rice at the seedling stage, to date, the underlying mechanisms responsible of salinity tolerance in *Saltol*-introgressed lines remain largely unknown.

Most QTLs so far identified for salinity tolerance in rice derive from *indica* accessions (e.g., Pokkali and Nona Bokra). More recently, genome-wide association mapping identified candidate QTLs for tolerance to mild salinity stress in temperate *japonica* rice (Batayeva et al., 2018; Frouin et al., 2018). *Saltol* introgression into *japonica* varieties was reported to improve salt tolerance in introgression lines (ILs) (Usatov et al., 2015). In other studies, however, the *Saltol* QTL from the *indica* cultivar IR64-*Saltol* was introgressed into a *japonica* rice variety, and no differences could be observed between *Saltol* and non-*Saltol* ILs (Han et al., 2020). When FL478, a RIL derived from the cross between IR29 and Pokkali, was used as a *Saltol* donor, some lines that introgressed only the *Saltol* QTL were found to exhibit less salinity tolerance than their donor parent, thus, suggesting the presence of regions in the FL478 genome other than the *Saltol* region contributing to salinity tolerance (Kim et al., 2009; Thomson et al., 2010; Alam et al., 2011). Clearly, many questions still remain unanswered regarding the use of the *Saltol* QTL in salinity tolerance in rice. Furthermore, considering that most QTLs so far identified for salinity tolerance are from *indica* subspecies, it is important to investigate the genome-wide impact on gene transcription in a recipient *japonica* background caused by introgressions of *indica* genomic regions. In this study we report the production and characterization of salt tolerant ILs derived from the cross of FL478 (*indica* cv, donor for the *Saltol* QTL), and the salt-sensitive elite *japonica* variety OLESA. Kompetitive allele specific PCR (KASPar) genotyping and genotyping by sequencing (GBS) were used to monitor the introgression of *indica* segments into OLESA and to further characterize advanced ILs. Evaluation of salt tolerance in the ILs was conducted at the seedling stage in hydroponically grown plants. A fine-mapping of *indica* introgressions was performed in the best performing IL (IL22) by transcriptome-based genotyping. Furthermore, we provide a global view of the leaf transcriptome of IL22 plants, both under control and salt-stress conditions. Evidence is presented that genes in the introgressed *indica* regions at Chromosome 1 (including *Saltol*) and Chromosome 3, in the IL22 line, might well contribute to a phenotype of salt tolerance, either by their intrinsic function (e.g., ion transporters and cell wall biosynthesis genes), or by functional interactions with other *indica* or *japonica* genes. The information gained in this study extend our understanding on the molecular mechanisms involved in salinity tolerance in introgression rice lines harboring the *Saltol* QTL which might also be useful for the identification of marker genes for salinity tolerance in rice.

## Materials and Methods

### Plant Material and Marker-Assisted Backcross Breeding

Introgression lines were generated through backcross breeding between FL478 (male donor, *indica* ssp.) harboring the *Saltol* QTL (Thomson et al., 2010) and OLESA, a salt sensitive, long grain *japonica* cultivar (female recipient) (Supplementary Figure 1). FL478 was obtained from the International Rice Research Institute (IRRI, accession number IRGC 117406). OLESA was provided by Càmara Arrossera del Montsià SCCL (Amposta, Spain, accession number NRVC20120346). It involved the initial cross and three backcrosses coupled to embryo rescue (Ohnishi et al., 2011), and three selfing steps. Complementary approaches were used to monitor the introgression of *indica* segments into the *japonica* background of OLESA. The first step consisted in the screening of progenies for the presence of the *Saltol* QTL using the *Saltol* linked SSR marker SKC10 (Thomson et al., 2010) which was found to be polymorphic between the two parents (FL478 and OLESA) (Supplementary Figure 2). The second genotyping step was carried out on positive samples for the SKC10 SSR marker and involved the analysis of *Saltol* single nucleotide polymorphism (SNP) flanking markers located at 9.06 and 13.34 Mb in Chromosome 1 using KASPar (Kompetitive allele specific PCR) coupled to Light Cycler 480 system technology (Roche) (Supplementary Table 1). Finally, KASPar coupled to the Fluidigm technology was used for foreground and background selection. A total of 68 SNPs distributed along the 12 chromosomes were assayed (Supplementary Table 1).

### Genotyping-by-Sequencing

Genomic DNA was extracted from young leaves of ILs and their respective parental lines by high-throughput automated methods using NucleoMag Plant kit (Macherey-Nagel) and its quality verified on 1.5% agarose gel. After Hoechst quantification, DNA’s concentrations were normalized. DNAs were digested individually with the *Ape*KI restriction enzyme. GBS sequencing library was prepared by ligating the digested DNA to unique nucleotide adapters (barcodes) followed by PCR. Sequencing was performed using Illumina HiSeq3000. To detect informative SNPs, fastq files were analyzed using Tassel V5 pipeline and an alignment on the *O. sativa* Nipponbare reference genome (MSU7) with Bowtie2. Only polymorphic parental *loci* were kept and filtered on heterozygous rate (<40%) and then imputed with Beagle v5.0.

### Phenotyping for Salinity Tolerance

Introgression lines derived from FL478 × OLESA (30 lines, BC_3_F_4_) were screened in hydroponic experiments using the FL478 and OLESA cultivars as tolerant and sensitive checks, respectively. Rice seeds were pregerminated in water for 7 days and then transferred to hydroponic tanks with modified Yoshida solution (1.43 mM NH_4_NO_3_, 0.51 mM K_2_SO_4_, 0.85 mM KH_2_PO_4_, 0.12 mM K_2_HPO_4_, 0.75 mM CaCl_2_⋅2H_2_O, 1.64 mM MgSO_4_⋅7H_2_O, 9.5 μM MnCl_2_⋅4H_2_O, 0.075 μM (NH_4_)_6_⋅Mo_7_O_24_⋅4H_2_O, 18.9 μM H_3_BO_3_, 0.15 μM ZnSO_4_⋅7H_2_O, 0.16 μM CuSO_4_⋅5H_2_O, 35.75 μM FeSO_4_-EDTA, pH 5) for 7 days. Salt treatment was applied by supplementing the nutrient solution with 80 mM NaCl for 14 days. Control plants were not supplemented with NaCl. No aeration was applied. Plants were randomly distributed, in a ratio of 40 plants per 10 L tank (5 and 10 plants per genotype, control and salt conditions, respectively). Plants were grown at 28/26°C day/night with a 14/10 h light/dark cycle. The nutrient solution was replaced weekly.

Fresh weight (FW), dry weight (DW), shoot length (SL), root length (RL), and SES score (standard evaluation system, IRRI) were examined at 14 days after salt treatment. FW, DW, SL, and RL were calculated as the percentage of FW, DW, SL, or RL in salt conditions compared to control conditions and then normalized to OLESA sensitive check. In this way, the improvement or worsening of salinity tolerance in ILs in comparison to its recurrent parent OLESA could be determined. Electrolyte leakage (EL) analyses of young leaves were carried out at 2 days after salt treatment as previously described (Campo et al., 2014). Briefly, leaves were cut in 1 cm segments and washed in Milli-Q water. Then, leaf segments were incubated for 2 h in Milli-Q water in a shaker and electro conductivity 1 (Ec1) was measured. After autoclaving the leaf segments for 20 min, Ec2 was measured and the EL was calculated as (Ec1/Ec2) × 100. Three independent experiments were performed (three replicates from four plants each). For Na^+^/K^+^ molar ratio evaluation, plants were harvested at 7 days of salt treatment. In order to remove apoplastic Na^+^ and K^+^, roots where washed twice for 10 min at 4°C in a 25 mM Rb_2_SO_4_ solution before dissecting the plant tissues (young leaves, old leaves, sheath and stem, and roots). Roots and aerial parts were air-dried and then mineralized by a microwave digester system (MULTIWAVE-ECO, Anton Paar GmbH) in 65% (v/v) HNO_3_. Aliquots of the mineralized samples were adequately diluted in Milli-Q water and the concentrations of Na^+^ and K^+^ were measured by ICP-MS technique (Bruker Aurora M90 ICP-MS, Bruker Daltonik GmbH). Three independent experiments were performed (three replicates from three plants each).

### RNA-Seq Library Preparation, Sequencing, and Data Analysis

Plants (IL22, OLESA) were grown in hydroponic cultures as described above (phenotyping for salinity tolerance). Leaves from salt-treated (80 mM NaCl, 24 h of treatment) and untreated control plants were harvested (three replicates per genotype and condition, four plants per replicate). Samples were immediately frozen in liquid nitrogen and grinded by TissueLyser (Qiagen). Total RNA was extracted using the Maxwell RSC Plant RNA Kit (Promega). Details of library preparation and RNA-Seq analysis are presented in Supplementary Method. Statistical analysis of read counts was performed with R, with the HTSFilter package to remove low-expressed genes and the edge R package. Criteria used for calling differentially expressed genes (DEGs) were fold-change (log_2_ fold change >0.5 or <−0.5, for up-regulated and down-regulated genes), significance of differences in expression (*P* ≤ 0.05), and expression level (fragments per kilobase per million mapped reads-FPKMs ≥ 25). Gene Ontology (GO) enrichment of DEGs was carried out by Singular Enrichment Analysis (SEA) using AgriGO (Tian et al., 2017). Data sets for RNA-Seq analyses have been deposited at the National Center for Biotechnology Information (NCBI) Gene Expression Omnibus (GEO) with accession number GSE167342).

### Transcriptome-Based Genotyping

Data sets obtained by RNA-Seq analysis were used for the fine mapping of *indica* regions introgressed into the genome of IL22 plants (*japonica* background). Details on the various steps followed for the identification of introgressed *indica* regions in IL22 plants are presented in Supplementary Method.

### Reverse Transcription-Quantitative PCR

Total RNA was obtained as described above (RNA-Seq library preparation and sequencing). Total RNA (1 μg) was retrotranscribed using the High Capacity cDNA reverse transcription Kit (Applied Biosystems, Thermo Fisher Scientific). *Reverse transcription-quantitative PCR* (RT-qPCR) analyses were carried out in 96-well optical plates in a LightCycler^®^ 480 System (Roche) according to the following program: 10 min at 95°C, 45 cycles of 95°C for 10 s and 60°C for 30 s, and an additional cycle of dissociation curves to ensure a unique amplification. The reaction mixture contained 5 μl of SYBR Green Master mix reagent (Roche), 2 μl of 1:4 diluted cDNA samples and 300 nM of each gene-specific primer in a final volume of 10 μl. The results for the gene expression were normalized to *OsUbi1*. Three biological replicates each one from a pool of four different plants, and three technical replicates for each biological replicate were analyzed. Primers were designed using Primer-BLAST^1^. Primers used for RT-qPCR are listed in Supplementary Table 2.

### Statistical Analysis

Significant differences in FW, DW, SL, and RL, EL, Na^+^/K^+^ ratio, and RT-qPCR experiments were determined by Student’s *t*-test, evaluating pairwise comparisons of mean differences. For RT-qPCR analysis, statistical significance was determined by Student’s *t*-test (salt-treated vs non-treated, each genotype).

## Results

### Development of Introgression Lines by Marker-Assisted Backcross Breeding

In this study, marker-assisted backcross (MABC) breeding was used to transfer the *Saltol* QTL from the highly salt tolerant *indica* RIL FL478 (male donor parent) into the salt-sensitive *japonica* elite cultivar OLESA (female recurrent parent). Three successive cycles of marker-assisted backcrossing coupled to embryo rescue (Ohnishi et al., 2011), followed by three cycles of selfing were carried out (Supplementary Figure 1). Several marker-assisted approaches were used for selection of progenies through the backcrossing process, which involved the use of *Saltol* SSR marker SKC10 by PCR (Thomson et al., 2010) and KASPar markers coupled to the Light Cycler 480 system and the Fluidigm technology (Roche) (Supplementary Figure 2 and Supplementary Table 1). A total of 30 BC_3_F_3_ inbred lines showing a recurrent parent genome (RPG) recovery ranging from 92.7 to 100% were identified (average of 96.9% RPG) which were advanced to BC_3_F_4_ by selfing. The ILs were all homozygous for the 68 SNPs used for foreground and background selection at the BC_3_F_3_ generation (Supplementary Figure 3). In addition to the *Saltol* region (Chromosome 1), these lines had additional introgressions of different lengths distributed in different chromosomes (Supplementary Figure 3). The 30 BC_3_F_4_ lines derived from four different BC_3_F_1_ plants, which resulted in four different introgression patterns in the ILs at the BC_3_F_3_ generation. Thus, depending on the pattern of *indica* introgressions, the ILs classified into four different groups (groups I, II, III and IV) (Supplementary Figure 3).

### Genotyping-by-Sequencing Genotyping of Introgression Lines

**FIGURE 1:**
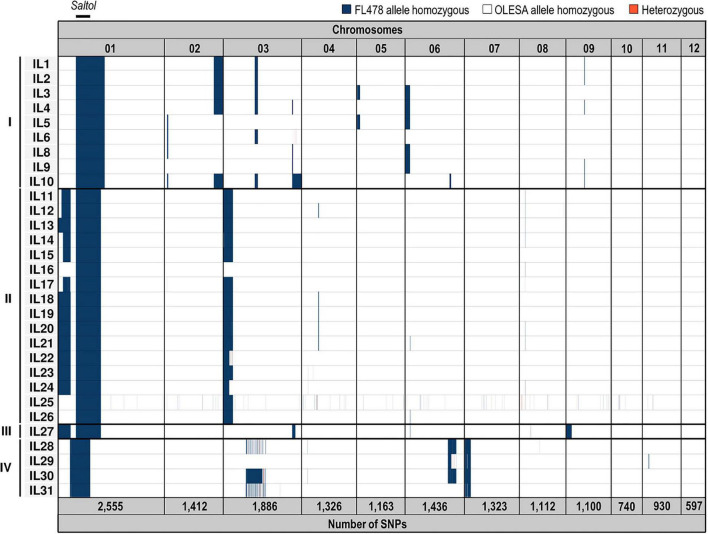
Genotyping-by-sequencing analysis of introgression lines. Scheme representing SNPs variation and its distribution among the 12 chromosomes in BC_3_F_4_ introgression lines. SNPs are indicated in columns according to their chromosomal location. Introgression lines (IL1 to IL31) are clustered in four groups (I to IV) depending on the BC_3_F_1_ parent from which they derive. The *Saltol* QTL location (and length) is indicated in the upper part. The total number of SNPs found in each chromosome is indicated in the lower part. Donor (FL478) and recurrent (OLESA) homozygous alleles in the 12 chromosomes are depicted in blue and white, respectively, and heterozygous in red. A detailed description of SNPs is provided in Supplementary Table 3.

Genotyping by sequencing (GBS) represents a powerful approach to detect genotypic variation, such as SNPs and insertion/deletions in crop species. In the present study, GBS was used to identify allelic variations between ILs generated by MABC breeding. A total of 15,580 SNPs were identified in the 30 ILs here investigated, its number being variable depending on the rice chromosome (Figure 1 and Supplementary Table 3). Polymorphic SNPs were not randomly distributed among all the 12 chromosomes, with the highest SNP number found on Chromosome 1 (*Saltol* QTL). A relatively low number of SNP polymorphisms were observed in Chromosomes 8, 10, 11, and 12. ILs genotyping obtained in the GBS data was in accordance with the genotypes observed using KASPar markers, showing the *Saltol* QTL completely introgressed in homozygosis in the 30 inbred lines with some other small introgressions in the other chromosomes. As previously observed by KASPar analysis, GBS confirmed four groups of *indica* introgression patterns (I to IV) derived from four different BC_3_F_1_ plants. Additionally, GBS analysis allowed us to identify allelic variations in the introgressions in lines grouped into the same group. The value added by seeing the allelic profile of markers more densely distributed on the whole genome, was in *a posteriori* consideration of the choice of the KASPar makers used for the introgression of *Saltol*. For instance, a large flanking region surrounding the target QTL and some areas on Chromosome 3 maintained a significant portion of the donor parents in more than half the progenies, despite the three cycles of backcrossing. Therefore, GBS allowed us to discover and call SNPs on 30 advanced inbred lines of rice for salinity tolerance, while defining common and specific polymorphic SNPs between the various ILs.

### Phenotypic Characterization of Introgression Lines

Introgression lines harboring the *Saltol* QTL in homozygosis (30 lines) were evaluated for salinity tolerance at the seedling stage in hydroponic cultures. Before assaying salt tolerance of ILs, the two parental lines were evaluated for salinity tolerance at three different NaCl concentrations, namely 60, 80, and 100 mM, using the Standard Evaluation System (SES; IRRI). The SES score rate the symptoms of salt stress on a scale of 1 to 9 based on morphological symptoms, where a lower score of SES indicates tolerance and a higher score indicates sensitivity to salt stress. Differences between the two parental lines were better seen using a NaCl concentration of 80 mM and a period of treatment of 14 days (Supplementary Figure 4).

**FIGURE 2:**
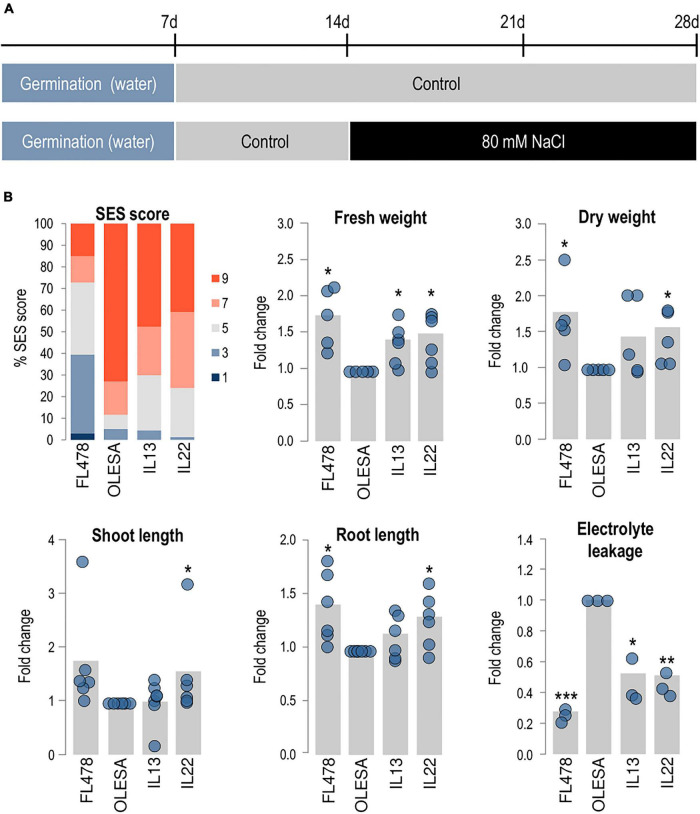
Characterization of salt tolerant introgression lines. Introgression (IL13, IL22) and parental (FL478, OLESA) lines were grown hydroponically in modified Yoshida solution and exposed to salt stress (80 mM NaCl). Student’s *t*-test (*P < 0.05, **P < 0.001, ***P < 0.0001). **(A)** Experimental design used to assess salinity tolerance. **(B)** Analysis of salinity tolerance at 14 days [SES scores, fresh weight (FW), dry weight (DW), shoot length (SL), and root length (RL)] or 2 days (electrolyte leakage, EL) after treatment. SES scores are shown as the percentage of plants at each score value. 1, highly tolerant; 3, tolerant; 5, moderately tolerant; 7, sensitive; 9, highly sensitive. Six independent experiments were carried out (10 plants/genotype each experiment). For FW, DW, SL, RL, and EL, values in each genotype were normalized to those in OLESA. Gray bars represent the fold change of each line in comparison to OLESA values, calculated by the means of six independent hydroponic cultures (blue dots) with 10 plants per genotype in each hydroponic assay (5 plants in control conditions). EL was measured in three independent experiments (12 plants/genotype each experiment).

Next, the ILs (30 lines) were evaluated for salinity tolerance in successive rounds of hydroponic assays (80 mM NaCl for 14 days; six independent assays) (Figure 2A). At the onset of salt treatment, all the plants were at the same developmental stage (two-leaf stage). In each assay, the most salt-sensitive lines were discarded. SES evaluation revealed different degrees of salt-induced damage among lines from the four groups (I to IV, according to the BC_3_F_1_ parent from which each line derived), as well as among lines in a given group (Supplementary Figure 5A). Thus, even though all the ILs were homozygous for the markers linked to *Saltol* (BC_3_F_4_ plants), there was an extensive variation in phenotypic responses to salt stress among the 30 ILs. For subsequent analyses, we focused on lines IL22 and IL13, these lines performing well in salt tolerance assays compared with the salt-sensitive parent OLESA (Supplementary Figures 5A,B). The ILs developed new leaves under salt stress (Supplementary Figure 6), and were able to flower and set seeds when the stress was removed (e.g., by transferring the plants from hydroponic cultures to soil).

Based on SES, 73.1% of the OLESA plants showed a SES score of 9 (highly sensitive), but only 40.9 and 47.8% of IL22 and IL13, respectively, scored in this category (Figure 2B). Also, 22.5 and 25.4% of the IL22 and IL13 plants, respectively, had a score of 5 (moderately tolerant), while only 6.4% of plants from the recurrent parent OLESA scored in this category. Analysis of morphological traits also indicated that lines IL22 and IL13 were the best performing ILs in salt tolerance assays (Figure 2B). Compared with the sensitive and tolerant parents (OLESA and FL478, respectively), the IL22 and IL13 lines showed intermediate responses in the various phenotypical parameters assayed (i.e., FW, DW, SL, and RL) (Figure 2B).

Then, we examined EL in leaves of IL plants, which was compared with that of their sensitive and tolerant parents. EL is an indicator of cell membrane injury and it is commonly used for the identification of salt tolerant plants (Bajji et al., 2002; Demidchik et al., 2014). As expected, upon exposure to salt stress, the parental varieties FL478 and OLESA (salt tolerant and salt sensitive, respectively) showed low and high levels of EL, respectively (Figure 2B). Compared with the sensitive parent, IL22 and IL13 exhibited reduced EL values, indicating less membrane damage during salt treatment (Figure 2B).

Maintenance of adequate Na^+^/K^+^ ratios in plant tissues under salt stress is essential for salinity tolerance, and *Saltol* has been found to be associated with low Na^+^/K^+^ ratio in rice seedlings (Gregorio, 1997; Bonilla et al., 2002; Gregorio et al., 2002; Waziri et al., 2016). Accordingly, we estimated the Na^+^/K^+^ molar ratio in different tissues of IL22 and IL13 plants (e.g., young leaves, old leaves, sheath and stem, and roots), as well as in the whole shoots (Table 1 and Supplementary Table 4). The Na^+^/K^+^ ratio in IL plants was compared with that in the parental varieties FL478 and OLESA. In roots, the Na^+^/K^+^ ratio of ILs and parent plants was similar (Table 1). Further, the Na^+^/K^+^ ratio in tissues, and in particular in the young leaves, of IL plants was significantly lower compared with the salt-sensitive parent (OLESA), indicating that these plants are less stressed at the cellular level than OLESA plants. Thus, values of Na^+^/K^+^ ratios in shoots of IL22 and IL13 plants were consistent with the observed phenotypic responses to salt treatment in these lines.

**TABLE 1:** Na^+^/K^+^ molar ratios in different tissues of introgression rice lines and parents.

	Young leaves	Old leaves	Sheath and stem	Shoot	Root
FL478	0.22 ± 0.00**	1.19 ± 0.17**	1.21 ± 0.18*	0.90 ± 0.12**	1.89 ± 0.06
OLESA	1.94 ± 0.18	2.92 ± 0.17	5.33 ± 0.57	3.29 ± 0.17	1.72 ± 0.17
IL13	1.01 ± 0.09**	2.07 ± 0.16*	2.53 ± 0.09*	2.11 ± 0.11*	1.85 ± 0.13
IL22	0.91 ± 0.18*	2.53 ± 0.06	3.22 ± 0.01*	2.41 ± 0.09*	1.90 ± 0.03

Data shows mean ± SEM in comparison to OLESA values (Student’s t-test, *P < 0.05, **P < 0.01).

Collectively, results obtained in salt tolerance assays revealed lower SES scores, reduced EL values and lower Na^+^/K^+^ ratios in leaves of IL22 and IL13 plants, thus, supporting enhanced salinity tolerance in these introgression rice lines.

### Fine Mapping of *Indica* Introgressions in IL22 by Transcriptome Genotyping

The observation that ILs exhibited extensive phenotypic variation in response to salt stress, even though all these lines harbor *Saltol* in homozygosis, raised interesting questions. We reasoned that *indica* introgressions might have an effect on the leaf transcriptome of the IL, not only because of the introgression of favorable *indica* genes from FL478, but also because epistatic interactions between *indica* introgressions and genes from the *japonica* background (OLESA) might occur. In this respect, salinity tolerance, and maintenance of low Na^+^/K^+^ ratios at the seedling stage, is known to be governed by both additive and dominant effects in rice (Gregorio and Senadhira, 1993). Understanding the overall impact of *indica* introgressions into the genetic background of *japonica*, however, requires knowing the precise *loci* introgressed that are transcriptionally active in a particular IL. The IL22 line performing best in salt tolerance assays was selected for these studies.

In this work, a transcriptome-based approach was used with a twofold objective: (i) to get a fine mapping of *indica* genetic introgressions in IL22, and (ii) to assess the expression of *indica* and *japonica* genes in IL22 plants. For this, IL22 and OLESA plants were grown in hydroponic culture under control and moderate salt stress conditions (80 mM NaCl, 24 h of salt treatment). Comparisons of RNA-Seq data from each genotype (IL22, OLESA) and condition (control, salt-treated) were made as shown in Supplementary Figure 7 (fine mapping of *indica* introgressions in IL22; comparative transcriptomes of IL22 and OLESA, control and salt stress conditions).

**FIGURE 3:**
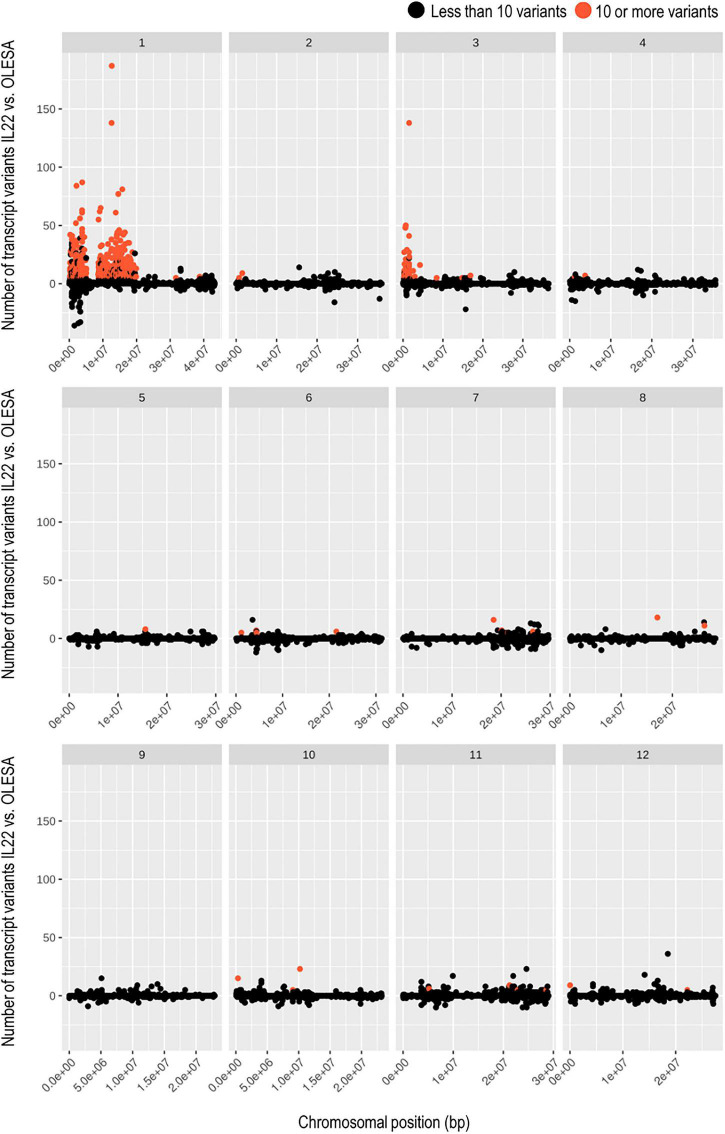
Transcriptome-based genotyping of the introgression line IL22. Scatter plots showing the number of transcript variants for each IL22 transcript with respect to the natural variation of OLESA transcripts (both compared to *japonica* reference genome). Transcripts identified as *indica* (red dots) are those showing 10 or more variants in IL22 than in OLESA. Transcripts showing less than 10 transcript variants in IL22 compared to OLESA were considered *japonica*. Each chromosome (1–12) show their scatter plot and transcripts are sorted by chromosomal position, indicated in the *x*-axis.

To get a fine mapping of the *indica* regions introgressed into the genome of IL22 plants, RNA-Seq reads from control and salt-treated plants were aligned to *japonica* (cv Nipponbare, IRGSP-1.0/Ensembl release 42) reference genome. As a control of natural variation, OLESA transcripts were also mapped against *japonica* reference genome. By comparing the number of transcript variants in IL22 and OLESA, three large genomic regions containing high frequency of transcript variants in IL22 (indicating the presence of *indica loci*) were identified: two large introgressions located in Chromosome 1, while the third one located at Chromosome 3 (Figure 3). These results were essentially in accordance with those obtained by GBS and KASPar genotyping of IL22. Most importantly, this analysis allowed us to identify the introgression boundaries for each introgressed region, hence, the precise *loci* contained in the various introgressed *indica* intervals. In Chromosome 1, a total of 1,213 genes were identified: 583 and 630 genes in Block 1 and Block 2, respectively, Block 2 containing the *Saltol* QTL (Supplementary Table 5). The *indica* region introgressed in Chromosome 3 contained 354 genes (Supplementary Table 5). Additionally, 28 *indica* genes distributed among other rice chromosomes, except Chromosome 9, were identified (Supplementary Table 5).

**FIGURE 4:**
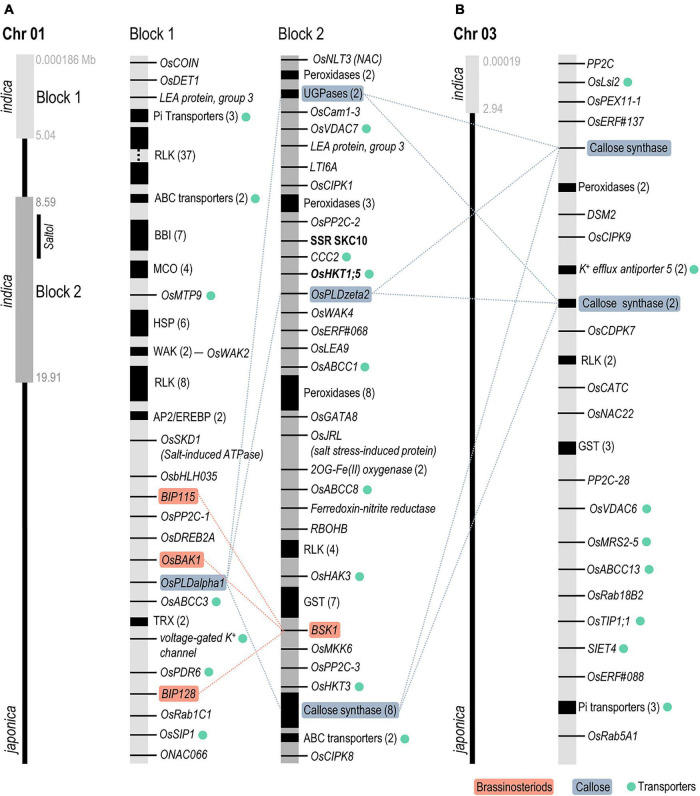
Scheme of *indica* introgressions identified in Chromosome 1 (**A**, Block 1 and Block 2) and Chromosome 3 **(B)** of IL22 plants. Black regions denote clusters of genes (the number of genes in each cluster is shown in parenthesis). *2OG-Fe(II) (2-oxoglutarate Iron 2), ABC* (*ATP Binding Cassette transporters*), *AP2/EREBP* (*APETALA2/ethylene-responsive element binding protein* TF), *BAK* [*Brassinosteroid Insensitive 1 (BRI1)-associated receptor kinase*], *BBI* (*Bowman–Birk proteinase Inhibitor*), *bHLH* (*Basic helix–loop–helix* TF), *BIP* [*Brassinosteroid receptor kinase (BRI1)-interacting protein*], *BSK1* (*BR-signaling kinase 1), Cam* (*Calmodulin*), *CATC* (*Catalase C*), *CCC2* (*Cation-Chloride cotransporter 2*), *CDPK7* (*Calcium-dependent protein kinase 7*), *CIPK* (*Calcineurin B-like interacting protein kinase*), *COIN* (*Cold inducible*), *DET1* (*De-Etiolated 1*), *DREB2A* (*Dehydration responsive element binding 2A*), *DSM2* (*drought-sensitive mutant 2*), *ERF* (*Ethylene Responsive Factor* TF), *GST* (*Glutathione-S-transferase*), *HAK (High-affinity Potassium Transporter*), *HKT* (*High-affinity Potassium Transporter*), *HSP* (*Heat Shock Protein*), *JRL* (*jacalin-related mannose-binding lectin*), *LEA* (*Late Embryogenesis Abundant protein*), *Lsi2* (*Low Silicon 2*), *LTI6A* (*Low temperature-induced 6A*), *MCO* (*Multicopper Oxidase*), *MKK6* [*mitogen activated protein kinase (MAPK) kinase 6*], *MRS2-5* (*Mitochondrial RNA Splicing, Mg^2+^ transporter 2-5*), *MTP9* (*Metal Tolerance Protein 9*), *NAC* (*NAM/ATAF/CUC* TF), *PDR6 (Pleiotropic Drug Resistance 6), PEX11-1* (*Peroxin 11-1*), *PLD* (*Phospholipase D*), *PP2C* (*Protein Phosphatase 2C*), *Rab (Ras-related in brain*), *RBOHB* (*Respiratory burst oxidase homolog B*), *RLK* (*Receptor-Like Kinase*), *SIET4* (*Silicon efflux transporter 4*), *SIP1* (*Small and basic intrinsic protein 1*), *SKCD1* (*Suppressor of K^+^ transport growth defect-like protein 1*), *TIP1;1* (*Tonoplast intrinsic protein 1;1*), *TRX* (*Thioredoxin*), *UGPases* (*UDP-glucose pyrophosphorylases*), *VDAC (voltage-dependent anion cannel*), and *WAK* (*Wall-Associated kinase*).

Singular enrichment analysis (SEA) of molecular functions using AgriGO (Tian et al., 2017) revealed enriched GO terms in *indica* regions introgressed into the IL22 genome. Genes in the category of “Protein kinase activity” and “Iron ion binding” were overrepresented in Chromosome 1 (Block 1 and Block 2, respectively). Genes in the “Iron ion binding” category included *peroxidase*, *Respiratory Burst Oxidase Homolog B* (*RBOHB*), *cytochrome P450*, *ferredoxin oxygenase*, as well as *protein phosphatase* genes (Supplementary Figure 8 and Supplementary Table 5). Regarding the *indica* region in Chromosome 3, this region was enriched in genes in the category of “Anion transmembrane transporter activity” (Supplementary Figure 8 and Supplementary Table 5). We also noticed that many genes in the introgressed *indica* regions were arranged in clusters (some examples are shown in Figure 4).

Interestingly, a large number of the genes identified in introgressed *indica* regions have been shown to play a role in salt and/or drought stress responses, such as those involved in ion homeostasis (e.g., Na^+^/K^+^ transporters) and water channel proteins (aquaporins) (Figure 4 and Supplementary Tables 5, 6). In addition to the high affinity HKT transporter *OsHKT1;5* (*SKC1* in the *Saltol* QTL), the *indica* region in Block 2 of Chromosome 1 also contained *OsHKT2.3/HKT3* and *OsHAK3* (putative K^+^ uptake transporters) (Figure 4). Other genes in *indica* regions of IL22 were those encoding salt-associated TFs (e.g., *DREB2A*), LEA proteins, antioxidant enzymes (e.g., Thioredoxins, Glutathione S-tranferases, and Peroxidases), and components of the salt stress-induced signal transduction pathways (e.g., calcium sensors, CIPKs, and CDPKs protein kinases and protein phosphatases) (Figure 4 and Supplementary Table 5). Finally, genes that function in cell wall biosynthesis and modification (xylogalactosyl transferases, expansins, callose synthases, etc.) were highly represented in the *indica* regions of IL22 (Supplementary Table 6).

One important finding of this study is that genes with related functions were present in the various *indica* regions in IL22. For instance, genes involved in brassinosteroid signaling were identified in the two *indica* segments in Chromosome 1. *Brassinosteroid Insensitive 1 (BRI1)-associated receptor kinase 1* (*OsBAK1*), and two *Brassinosteroid receptor kinase (BRI1)-interacting protein* genes (*OsBIP115, OsBIP128*) were present in Block 1 of Chromosome 1, while *BR-signaling kinase 1* (*BSK1*) was found in Block 2 of this chromosome (Figure 4 and Supplementary Table 5). Genes involved in modulation of ABA signaling pathway and ABA biosynthesis controlling tolerance to salt stress were also present in *indica* regions introgressed in IL22 (e.g., the NAC22 TF in Chromosome 3; OsDET1 in Chromosome 1) (Supplementary Table 5). Ethylene-related genes were also identified in *indica* regions in IL22 (Supplementary Table 5). Another example of genes with related functions identified in *indica* introgression refers to genes involved in callose deposition, such as *Phospholipase D* (PLD) in Block 1 (*PLDa1*) and Block 2 (*PLDz2*) of Chromosome 1, respectively, and *callose synthase* in Chromosome 1 (Block 2) and Chromosome 3 (Figure 4). Phospholipase D has been shown to regulate callose deposition in response to salt stress (Hunter et al., 2019). There is then the possibility that *indica* genes in different introgressed genomic regions might function in concerted action for salinity tolerance at the seedling stage in IL22 plants. They include genes related to hormone signaling, such as ABA, ethylene, or brassinosteroid signaling. In this respect, sophisticated crosstalk is known to occur among the different hormones during adaptation to salt stress in plants, including rice, with cooperative or antagonistic relationships between them (Yu et al., 2020). In particular, a number of studies revealed cross-talk between brassinosteroids and ABA in Arabidopsis and rice (Hu and Yu, 2014; Dong et al., 2020).

As for TF genes, previous studies investigated expression dynamics of genes encoding TFs that localize within the *Saltol* QTL (named as SalTFs) in contrasting genotypes of rice under control and salt stress conditions (Nutan et al., 2017). In that study, the authors demonstrated that SalTFs are differentially regulated in contrasting genotypes, these TFs also showing unique features in relation to their response to salt stress and during plant growth and development.

**FIGURE 5:**
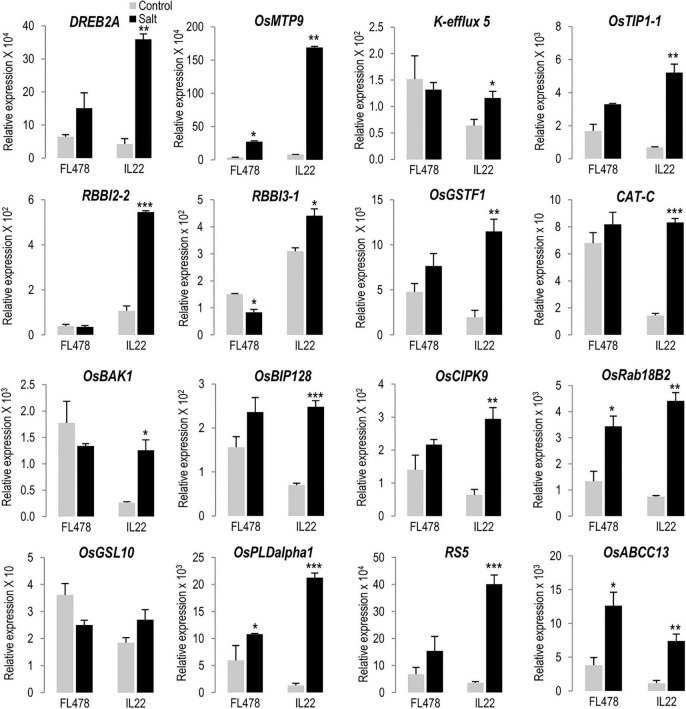
Salt-responsiveness of genes identified in *indica* regions introgressed in IL22. Plants were grown in hydroponic cultures under control and salt-conditions (24 h of salt treatment, 80 mM NaCl). Transcript levels of the indicated genes were determined by RT-qPCR. *OsUbi1* was used as the reference gene. Bars represent means of three biological replicates, each one from a pool of four different plants, ±SEM (Student’s *t*-test, *P ≤ 0.05, **P ≤ 0.005, ***P ≤ 0.0005; salt-treated vs un-treated plants). *DREB2A* (*Dehydration responsive element binding 2A*), *OsMTP9* (*Metal Tolerance Protein 9*, manganese transporter), *K^+^ efflux 5* (*K^+^ antiporter efflux 5*), *OsTIP1;1* (*Tonoplast intrinsic protein 1-1*), *RBBI2-2* (*Bowman–Birk proteinase Inhibitor 2-2*), *RBBI3-1* (*Bowman–Birk proteinase Inhibitor 3-1*), *OsGSTF1* (*Glutathione-S-transferase F1*), *CAT-C* (*Catalase C*), *OsBAK1* [*Brassinosteroid Insensitive 1 (BRI1)-associated receptor kinase 1*], *OsBIP128* [*Brassinosteroid receptor kinase (BRI1)-interacting protein 128*], *OsCIPK9* (*Calcineurin B-like interacting protein kinase 9*), *OsRab18B2* (*Ras-related in brain 18 B2*), *OsGSL10* (*Glucan Synthase-Like 10, callose synthase 10*), *OsPLDalpha1* (*Phospholipase D alpha 1*), *RS5* (*Raffinose synthase 5*), *OsABCC13* (*ATP Binding Cassette transporter B 13*). Primers and locus ID are listed in Supplementary Table 2.

Genes identified in *indica* regions of IL22 were arbitrarily selected and their expression analyzed by RT-qPCR analysis. They included genes encoding proteins involved in salt stress tolerance (i.e., TF DREB2A, K^+^ efflux 5 transporter, tonoplast intrinsic protein TIP1;1, and Calcineurin B-Like Protein-Interacting Protein Kinase 9-CIPK9) (Liu et al., 1994; Dubouzet et al., 2003; Li et al., 2008; Mallikarjuna et al., 2011; Zhu et al., 2018; Kurowska, 2021; Shabala et al., 2021). RT-qPCR analysis confirmed that *indica* genes are expressed in leaves of IL22 plants, and also revealed differences in the expression level of these genes between IL22 and its salt-tolerant *indica* parent FL478 when grown under control conditions (Figure 5, gray bars). Thus, under control conditions, some of these genes were found to be expressed at either a higher level, or a lower level, in IL22 plants compared with the salt tolerant parent FL478 (Figure 5, gray bars). Knowing this, we considered the possibility that the expression of salt-associated genes might have a differential regulation in IL22 plants (e.g., stronger induction) under salt stress conditions, as opposed to being constitutively up-regulated as in FL478. Supporting this possibility, salt-associated genes exhibited stronger induction in response to salt treatment in IL22 plants than in FL478 plants (e.g., salt-treated vs non-treated conditions) (Figure 5, black bars). Some examples are: *OsDREB2A* (an important regulatory TF controlling salt stress responses), *Cation efflux protein OsMTP9, aquaporin OsTIP1;1, glutathione-S-transferase OsGSTF1, Catalase CAT-C, Brassinosteroid receptor kinase (BRI1)-interacting protein OsBIP128, Calcineurin B-like interacting protein kinase OsCIPK9, Ras-related in brain 18B2 RAB18B2, Phospholipase PDLalpha1 OsPLDalpha1*, and *raffinose synthase 5 RS5*. There were also examples of genes that were induced by salt treatment in IL22, but not in FL478 (*K^+^ efflux transporter 5, Bowman–Birk proteinase inhibitor 2-2 RBBI2-2*), as well as genes showing an opposite regulation by salt stress in IL22 and FL478 plants [*Bowman–Birk proteinase inhibitor 3-1 RBBI3-1*, *Brassinosteroid Insensitive 1 (BRI1)-associated receptor kinase 1 OsBAK1, Glucan Synthase-Like 10 GSL10* also known as *callose synthase* 10]. Differences observed in the expression of *indica* genes among IL22 and FL478 suggest that regulation of salt stress responses might be distinctly different in IL22 relative to FL478 plants.

Collectively, these results suggested that *indica* regions introgressed in IL22 were enriched in salt-associated genes with different functions and that these genes, very likely, contribute to salinity tolerance in IL22 plants at the seedling stage. These genes might function either alone or in combination with other *indica* and *japonica* genes.

### Comparative Transcript Profiling of the Salt Tolerant Line IL22 and Its Salt-Sensitive Parent

**FIGURE 6:**
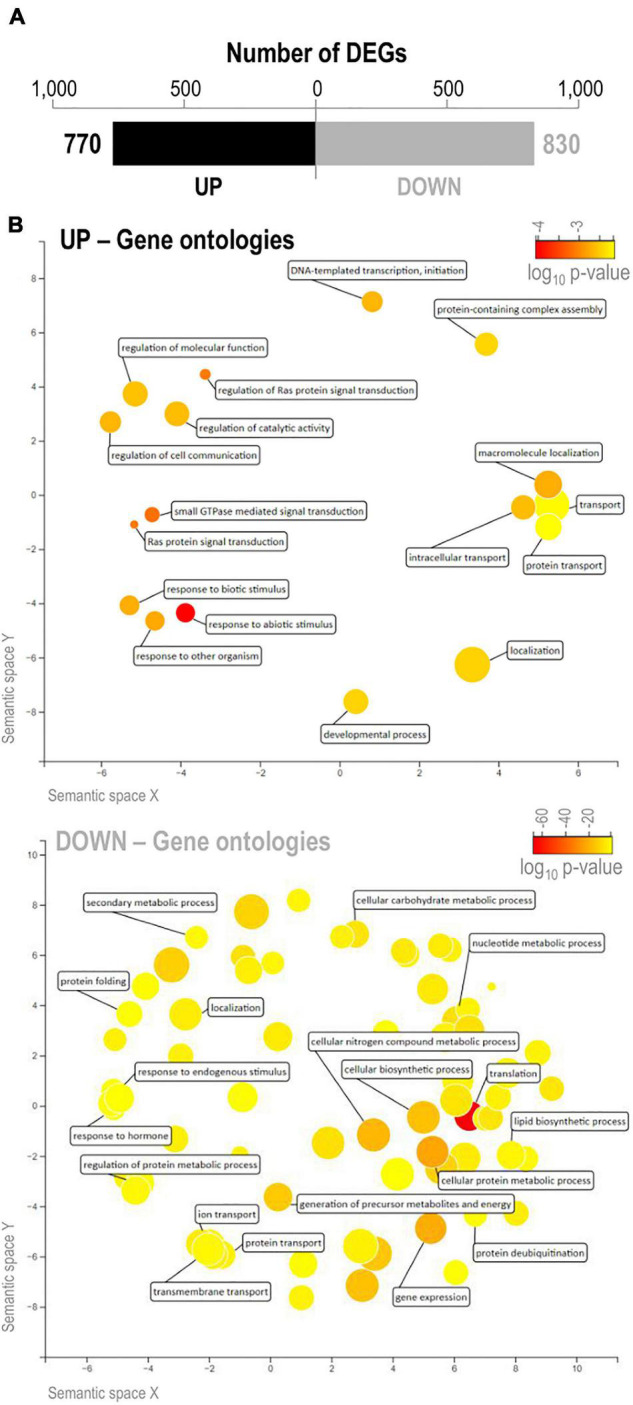
Differentially expressed genes in leaves of IL22 plants relative to OLESA plants. Plants grown in hydroponic culture under control conditions as indicated in Figure 2. Leaves of 2-week-old plants were used for RNA-Seq analysis. **(A)** Number of DEGs (black, up-regulated genes; gray, down-regulated genes). **(B)** Biological processes altered in IL22 plants relative to OLESA plants. GO terms enriched in up- and down-regulated genes in IL22 plants were visualized by REVIGO (Supek et al., 2011). The most enriched GO terms in the set of up-regulated and down-regulated genes are presented (upper and lower panels, respectively). Circles represent GO terms and those clustered closer to each other indicate similar GO terms. Color scale (yellow to red) represents the degree of GO enrichment (log_10_ *P*-value). Larger and smaller disc size represent more general and more specific terms, respectively.

To obtain further insights into the molecular mechanisms underlying salinity tolerance in IL22, we compared the leaf transcriptome of IL22 and OLESA plants grown under control conditions in both cases. We focused on transcriptional alterations in *loci* not included in the introgression intervals in IL22 plants (hereafter *japonica* genes), whose expression was compared with that of the corresponding genes in the salt-sensitive *japonica* parent OLESA. For calling DEGs, a log_2_ fold change (FC) >0.5 or <−0.5, and a *P*-value ≤ 0.05 were applied. In this way, 770 and 830 genes were found to be up-regulated and down-regulated, respectively, in IL22 compared with the salt-sensitive parent OLESA (Figure 6A and Supplementary Table 7). GO enrichment in biological processes was performed using AgriGO in up-regulated and down-regulated genes in IL22 plants, and visualized through the REVIGO tool (Supek et al., 2011). The GO category “Response to Abiotic Stimulus” was specifically enriched in up-regulated genes of IL22, though no salt treatment was applied to these plants (Figure 6B, upper panel and Supplementary Table 7). Other GO categories highly represented in up-regulated genes in IL22 plants were those related to “Transport,” “Transcription,” “Small GTPase-mediated signal transduction,” and “Regulation of Ras protein signal transduction” (Figure 6B, upper panel and Supplementary Table 7). Genes down-regulated in IL22 plants grouped into diverse categories with “Translation” being the dominant term in this set of genes (Figure 6B, lower panel and Supplementary Table 7). Other genes down-regulated in IL22 plants categorized in “Gene expression,” “Protein deubiquitination,” “Transport,” and “Metabolic Processes” (Figure 6B, lower panel and Supplementary Table 7). Together, these results demonstrated that, in the absence of salt stress, the *indica* introgressions had a high impact on the global *japonica* leaf transcriptome of IL22 plants.

**FIGURE 7:**
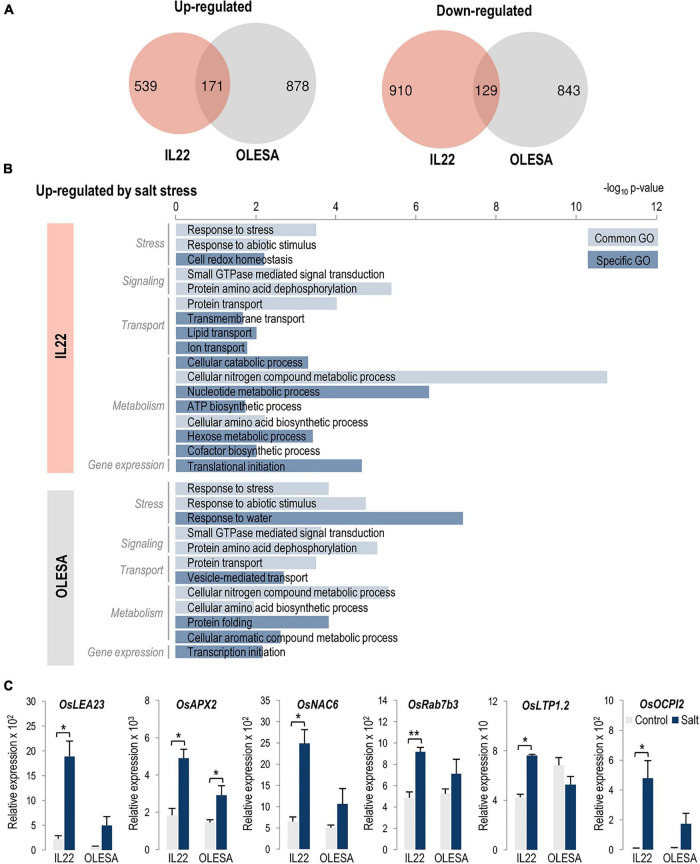
Comparison of OLESA and IL22 transcriptomes in response to salt stress (80 mM NaCl, 24 h of treatment). **(A)** Venn diagrams indicate the number of genes that are specifically and commonly regulated by salt stress in each genotype, up-regulated and down-regulated genes (log_2_ fold change >0.5 or <–0.5; *P*-value ≤ 0.05). **(B)** Enriched GO terms in biological processes in the up-regulated genes in each genotype performed using AgriGO (Tian et al., 2017). Light and dark blue bars indicate common and genotype-specific GO terms, respectively. The scale shows the statistical significance (–log_10_ *P*-value). **(C)** Salt-responsiveness of genes identified in *japonica* regions of IL22 and OLESA. Plants were grown in hydroponic cultures under control and salt-conditions (24 h of salt treatment, 80 mM NaCl). Transcript levels of the indicated genes were determined by RT-qPCR. *OsUbi1* was used as the reference gene. Bars represent means of three biological replicates, each one from a pool of four different plants, ±SEM (Student’s *t*-test, *P ≤ 0.05, **P ≤ 0.005). *OsLEA23* (*Late Embryogenesis Abundant protein 23*), *OsAPX2* (*Ascorbate peroxidase 2*), *OsNAC6* (*NAM/ATAF/CUC 6* TF), *OsRab7b3* (*Ras-related in brain 7 B3*); *OsLTP1.2* (*Lipid transfer protein 1.2*), and *OsOCPI2* (*Oryza sativa chymotrypsin inhibitor-like 2*). Primers and locus ID are listed in Supplementary Table 2.

Using the same criteria described above (log_2_ FC >0.5 or <−0.5, *P*-value ≤ 0.05), we investigated differences in the leaf transcriptome of IL22 and OLESA plants under moderate salt stress conditions (80 mM NaCl, 24 h; see Supplementary Figure 7). Pair-wise comparisons of the leaf transcriptional response to salt treatment with their respective controls revealed 1,749 and 2,021 genes as differentially expressed in IL22 and OLESA plants, respectively (Figure 7A and Supplementary Table 8). Notably, a relatively low number of genes were found to be commonly regulated in the two genotypes (171 and 129 genes, up- and down-regulated, respectively) suggesting that there is a genotype-specific response to salt treatment in an important number of genes (Figure 7A). GO terms in Biological Processes specifically enriched in the set of up-regulated genes in IL22 plants were: “Cell redox homeostasis,” “Transmembrane transport,” “Transport” (Lipid and Ion transport), Metabolic Processes (“Cellular catabolic processes,” “Nucleotide metabolic process,” “ATP biosynthetic process,” “Hexose metabolic process,” and “Cofactor biosynthetic process”), and “Translation initiation” (Figure 7B and Supplementary Figure 9). Biological Process terms specifically enriched in up-regulated genes in OLESA plants were “Response to water,” “Vesicle-mediated transport,” “Protein folding,” “Cellular aromatic compound metabolic process,” and “Transcription initiation” (Figure 7B and Supplementary Figure 10). Finally, genes that were down-regulated by salt stress classified into a broad spectrum of Biological Processes in both genotypes (IL22 and OLESA) (Supplementary Figures 11, 12). Gene expression correlation between RNA-Seq and RT-qPCR analyses in IL22 and OLESA plants could be observed for selected genes (Figure 7C).

For better understanding of salt-responsive mechanisms in IL22 plants, all salt-responsive genes of IL22 and OLESA plants were visualized using the MapMan (Version 3.6.0RC1) software. This analysis showed that the salt stress response mechanisms were complex and involved multiple mechanism and signal transduction pathways while confirming as a differential regulation of salt responses in genes in multiple processes. For example, multiple genes related to hormone signaling, transcriptional regulation, stress responses or ion transport, among others, were differentially regulated in IL22 and OLESA plants (Supplementary Figure 13 and Supplementary Table 8). This differential regulation of salt stress responses might well be the consequence of interactions between introgressed *indica* genes and genes in the recipient genome of IL22. Additional studies are, however, needed to understand gene regulatory networks operating in IL22 plants.

Collectively, comparative transcriptome analysis of IL22 and OLESA plants revealed that introgression of *indica* genes in IL22 is accompanied by a wide-range of alterations in gene expression under non-stress and salt stress conditions. The expression of genes involved in ion/water transport, redox homeostasis, protein phosphorylation/dephosphorylation processes, lipid transport, transcriptional regulation, hormone signaling, as well as genes involved in diverse metabolic processes showed differential regulation in IL22 plants relative to its salt-sensitive parent OLESA.

## Discussion

In this work, MABC breeding and step-wise phenotypic selection in hydroponic culture was used for the generation of salt tolerant ILs derived from crosses between the salt tolerant *indica* FL478 and the salt sensitive *japonica* OLESA. Based on phenotypic analyses (fresh weight, dry weight, shoot length, and root length) and salt injury symptoms (SES score), two ILs (IL22 and IL13) were identified as the best performing lines under salt stress conditions. Compared with the recurrent parent OLESA, tolerance to salt stress in IL22 and IL13 plants correlated with reduced leaf EL, and indicator of reduced membrane injury, and lower Na^+^/K^+^ ratios in shoot tissues, which is also a hallmark of salinity tolerance in rice plants (Gregorio and Senadhira, 1993). Presumably, the introgression of favorable genes from the donor FL478 would cause a reduction of Na^+^/K^+^ ratios in leaves, which would alleviate the cytotoxic effects of Na^+^ during salt stress.

An important phenotypic variation was observed among inbred lines that are homozygous for *Saltol*, which can be explained by the different patterns of *indica* introgressions (as revealed by KASPar and GBS genotyping). Variation in salinity tolerance among ILs is consistent with the complexity of the genetic control of salinity tolerance in plants, a process in which a plethora of genes distributed through the rice genome are expected to be implicated. It is worth mentioning that, despite the *Saltol* QTL has been extensively employed for improvement of salinity tolerance in rice breeding, the specific *loci* in *Saltol* responsible for salinity tolerance is still a matter of debate (Walia et al., 2005; Cotsaftis et al., 2011; Li et al., 2018; López-Cristoffanini et al., 2020). So far, only *OsHKT1;5* located in the *Saltol* QTL has been shown to play a role in conferring salinity tolerance at the seedling stage (Kobayashi et al., 2017). In other studies, however, no significant differences in salinity tolerance could be observed between *Saltol*-containing rice lines and non-*Saltol*-containing backcross lines (Alam et al., 2011; De Leon et al., 2017; Han et al., 2020).

Results presented on the fine mapping and transcriptome analysis of IL22 demonstrated that introgression of large *indica* segments from FL478 in Chromosomes 1 and 3 of the *japonica* cultivar OLESA has important implications in salinity tolerance. A variety of genes with a function in salinity tolerance were identified in the introgressed *indica* regions (e.g., genes involved in signaling, ROS detoxification, ion transport, etc.). This information will help in the identification of novel genotype-transcriptome-phenotype correlations in tolerance to salt stress in rice. Since the plant response to salt stress is physiologically and genetically complex, genes in the *Saltol* genomic region, and genes located outside the *Saltol* region but interacting with genes in *Saltol*, are expected to be implicated in salinity tolerance in rice (Thomson et al., 2010; Nutan et al., 2017).

### Fine Mapping of the Genomic Introgressions in the Salt-Tolerant Line IL22

Transcriptome genotyping allowed us to know the exact number and identity of *indica* genes introgressed in IL22 plants. This study also allowed us to characterize the behavior of introgressed *loci* in their new *japonica* genomic background, thus, supporting the usefulness of RNA-based genotyping for the analysis of introgression rice lines. *Loci* contained in introgressed segments at Chromosomes 1 and 3 included genes related to: (i) ion (Na^+^, K^+^) and water transport (aquaporins), and plasma membrane- or tonoplast-localized H^+^-ATPses; (ii) production of H_2_O_2_ and ROS detoxification; (iii) hormone signaling (e.g., ABA and brassinosteroid pathways); (iv) signal transduction, such as protein kinases (CDPK7, CIPKs) and protein phosphatase PP2C; (v) transcriptional regulation (e.g., DREB2A); and (vi) salt-responsive genes [e.g., LEAs (*OsLEA9*) and Heat Shock proteins (HSPs)]. These genes are expected to contribute to the phenotype of salinity tolerance in IL22 plants. For instance, *indica* regions in IL22 contained an important number of ion transporter genes with a function in salt stress tolerance, such as Na^+^, K^+^, and Cl^–^ transporters (Van Zelm et al., 2020). The introgressed *indica* regions in IL22 also contained phosphate transporters and metal transporters, but the functional relevance of these transporters in salinity tolerance remains to be investigated.

**FIGURE 8:**
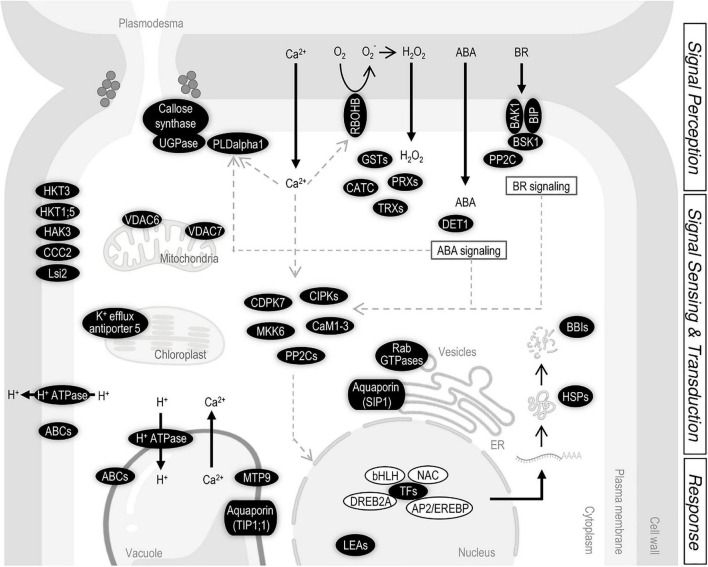
Summary of the salt-related genes located in the *indica* introgressed regions of IL22. Thick black arrows indicate entrance or exit of molecules. Thin black arrows indicate steps in a process or chemical reaction. Gray arrows indicate interactions or regulations between components. *ABC* (*ATP Binding Cassette transporters*), *AP2/EREBP* (*APETALA2/ethylene-responsive element binding protein* TF), *BAK1* (*Brassinosteroid Insensitive 1 (BRI1)-associated receptor kinase1), BBI* (*Bowman–Birk proteinase Inhibitor*), *bHLH* (*Basic helix–loop–helix* TF), *BIP* [*Brassinosteroid receptor kinase (BRI1)-interacting protein*], *BSK1* (*BR-signaling kinase 1), CaM1-3* (*Calmodulin 1-3*), *CATC* (*Catalase C*), *CCC2* (*Cation-Chloride cotransporter 2*), *CDPK7* (*Calcium-dependent protein kinase 7*), *CIPK* (*Calcineurin B-like interacting protein kinase*), *DET1* (*De-Etiolated 1*), *DREB2A* (*Dehydration responsive element binding 2A*), *GST* (*Glutathione-S-transferase*), *HAK3* [*High-affinity Potassium(K^+^) Transporter 3*], *HKT* (*High-affinity Potassium Transporter*), *HSP* (*Heat Shock Protein*), *LEA* (*Late Embryogenesis Abundant protein*), *Lsi2* (*Low Silicon 2*), *MKK6* [*mitogen activated protein kinase (MAPK) kinase 6*], *MTP9* (*Metal Tolerance Protein 9*), *NAC* (*NAM/ATAF/CUC* TF), *PLDalpla1* (*Phospholipase D alpha 1*), *PP2C* (*Protein Phosphatase 2C*), *PRX* (*Peroxidase*), *Rab GTPase (Ras-related in brain GTPase*), *RBOHB* (*Respiratory burst oxidase homolog B*), *SIP1* (*Small and basic intrinsic protein 1*), *TIP1;1* (*Tonoplast intrinsic protein 1;1*), *TRX* (*Thioredoxin*), *UGPAse* (*UDP-glucose pyrophosphorylase*), and *VDAC (voltage-dependent anion cannel*). Scheme created with BioRender.com.

Moreover, *indica* regions in IL22 included genes involved in the apoplastic production of H_2_O_2_ (*NADPH oxidase*) and ROS homeostasis, and regulatory genes in brassinosteroids (BRs) and ABA signaling (Figure 8). In Arabidopsis, it has been shown that H_2_O_2_ mediates the crosstalk of BRs and ABA pathways (Zhou et al., 2014; Tian et al., 2018). Equally, a role of BRs in salinity tolerance and connections between BRs signaling, ABA signaling, and ROS homeostasis have been described in rice (Sharma et al., 2013, 2017; Gui et al., 2016; Kaur et al., 2016).

Membrane remodeling has been documented in plants exposed to salinity (Guo et al., 2019). In Arabidopsis, it is known that the salt-induced increase in cytoplasmic Ca^2+^ concentration activates PLDα1 for phosphatidic acid production which, in turn, causes alterations in membrane properties (Hunter et al., 2019; Figure 8). In line with this, Phospholipase D (PLD) genes were identified in the introgressed *indica* regions in IL22, these genes potentially contributing to salinity tolerance in these plants.

The plant response to salt stress also involves cell wall modification which can benefit the plant by preventing water loss and modulating ion transport pathways. Along with this, the introgressed *indica* regions in IL22 contained many cell wall-related genes (see Supplementary Table 6). The set of *indica* genes in IL22 also included the two key genes responsible of callose biosynthesis, namely UDP-glucose pyrophosphorylase and callose synthase. Callose deposition at plasmodesmata is known to occur in salt-stressed Arabidopsis plants for the regulation of plasmodesmal permeability (Hunter et al., 2019). As it was observed for genes related to BRs signaling, genes involved in callose biosynthesis are located in independent introgression regions. This fact points to the possibility of interactions between *indica* genes in conferring salinity tolerance in IL22 plants.

An overview of genes identified in the *indica* segments in IL22, and the regulatory networks in which these genes participate in the plant response to salt stress, is presented in Figure 8. Based on the information available on mechanisms involved in salinity tolerance in plants, it can be hypothesized that salinity perception would trigger an increase in intracellular Ca^2+^ in IL22 plants that would then be decoded by Ca^2+^ sensors and Ca^2+^–dependent protein kinases for the activation of downstream protein phosphorylation/dephosphorylation cascades (e.g., genes encoding CaM1-3, CIPKs, CDPK7, MKK6, and PP2C) (Figure 8). Ca^2+^ is also a key regulator for PLD activity, and the PLDalpha1 is known to be involved in membrane lipid modification for the regulation of plasma membrane and function. Perception of salt stress would also trigger ROS production through the activity of the plasma membrane-localized RBOHB in IL22 plants (Figure 8). In turn, IL22 plants would activate the expression of enzymatic antioxidant systems to protect themselves from excessive levels of ROS (e.g., *CAT, PRX, GST*, and *TRX* genes) (Figure 8). Furthermore, CDPKs have been shown to regulate the production of ROS in plants (Kobayashi et al., 2007). Presumably, ion transporters (Na^+^ and K^+^ transporters) and water transporters identified in *indica* regions of IL22 plants that are known to localize at the plasma membrane, tonoplast, mitochondria or chloroplast, would help in maintaining appropriate cellular Na^+^ concentrations in IL22 plants (e.g., KHT1;5, HKT3, HAK3, Aquaporins, H^+^-ATPase and VDACs, among others) (Figure 8). Moreover, UGPase and callose synthase which are key genes in callose deposition at plasmodesmata were identified in *indica* regions, and expressed, in IL22 plants (Figure 8). Then, a regulation of the symplastic transport of ions, and other molecules, can be expected which might confer good adaptability to salt stress in IL22.

Importantly, a large number of TFs were identified in *indica* introgressed regions in IL22, of which *OsDREB2A* might have important implications for salinity tolerance. Constitutive expression of *OsDREB2A* in rice was reported to confer salt stress tolerance (Mallikarjuna et al., 2011). *ONAC022* (a NAC family member) and *OsCOIN* (a member of the zinc finger TF family) were also identified among *indica* genes in IL22 (Supplementary Table 5). The overexpression of these TFs has been shown to improve salt tolerance in rice (Liu et al., 2007; Hong et al., 2016). Moreover, *ONAC022*-overexpressing rice plants exhibited up-regulation of *OsDREB2* and *OsbZIP23* (Hong et al., 2016). As for *OsbHLH035* (also identified among *indica* genes in IL22), it was reported that this TF mediates seedling recovery after salt stress (Chen et al., 2018). Moreover, the *Saltol* QTL-localized TF *OsGATA8* was reported to be essential for salinity tolerance in rice seedling (Nutan et al., 2020).

Transcriptome analyses confirmed that *indica* genes in IL22 are expressed in the new genomic background. Different expression patterns of *indica* genes were, however, found between IL22 and its salt tolerant parent FL478, both under non-stress and salt stress conditions. These observations point to an effect of *japonica* background genes on the expression of introgressed *indica loci*. The basal expression of salt-associated *indica* genes in IL22 plants in the absence of salt stress may help IL22 plants to cope more effectively with subsequent salt stress. Furthermore, the observed superinduction of *indica* genes in IL22 plants suggest that these plants might be in a “priming status,” a mechanism that plants use to mount effective defense responses to pathogen attack (Martinez-Medina et al., 2016). Priming of salt-associated genes would help the plants to cope with a situation of salt stress. Likely, the expression of *indica* genes identified in introgression segments, either individually or in combination with other *indica* or *japonica* genes, would contribute to improve salinity tolerance in IL22 plants. On this point, an interesting finding of our study was the observation that two *NAC6* genes, *OsNAC6* and *SNAC1* (similar to *OsNAC6*) were induced by salt treatment in both IL22 and OLESA, but their expression reached a higher level in salt-treated IL22 than in salt-treated OLESA plants (*OsNAC6*, FC = 2.20 and 1.52 in IL22 and OLESA, respectively; *SNAC1*, FC 2.56 and 2.19 in IL22 and OLESA, respectively) (Supplementary Tables 8A,B). Furthermore, *OsZIP23* was among the set of salt-responsive genes in IL22 (FC = 2.20), but not in the set of salt-responsive OLESA genes. For *OsNAC6* and *OsZIP23*, a positive role in regulating the rice response to salt stress was previously reported (Nakashima et al., 2007; Xiang et al., 2008).

On the other hand, information available in the literature indicates the presence of retro-transposons and transposons within the *Saltol* QTL (Soda et al., 2013; Waziri et al., 2016). The presence of transposons in this region might well affect the expression of *indica* genes in the salt tolerant ILs. In particular, a MITE has been found to localize upstream of *OsHKT1;5*, and deletion of this MITE using the CRISPR/Cas9 system results in reduced *OsHKT1;5* expression (Wang et al., 2020). Finally, in this study, the RNA-Seq method was not used for the fine mapping of other salt-tolerant ILs. Further research is, however, needed to identify common and specific gene expression patterns among the various ILs and to correlate genomic and phenotypic data of ILs.

### Effect of *Indica* Introgressions on the Salt-Responsive Transcriptome of IL22 Plants

Currently, limited efforts have been performed to identify *Saltol* QTL x background interactions contributing to salinity tolerance. For instance, it is still unclear which are the effects of the *Saltol* introgression into the transcriptome of a recipient genome, or which genes within each genetic background (donor and recipient genomes) should be combined to effectively increase salinity tolerance. Results here presented revealed differences in the transcriptome of IL22 plants beyond the expression of *loci* in the introgressed *indica* segments, not only under control conditions, but also under moderate salt stress conditions in comparison to the salt-sensitive parent OLESA. We showed that a small number of genes are commonly regulated (up-regulated or down-regulated) by salt stress in the IL22 and OLESA plants, supporting a genotype-specific salt-responsive expression pattern in IL22 plants caused by *indica* introgressions. For instance, the set of salt-responsive genes in IL22, but not in OLESA, was enriched in genes involved in cell redox homeostasis and transport (i.e., transmembrane, ion, and lipid transport). Differences in the salt-responsive transcriptome of IL22 relative to OLESA also suggest that the expression of genes in the *indica* introgressed regions of IL22 has an important impact on the landscape of *japonica* genes from the recipient parent. Clearly, interactions between *indica* and *japonica* genes might well be responsible for the creation of novel expression profiles in IL22 plants and/or the modification of signaling pathways and regulatory networks involved in salinity tolerance.

Phytohormones, besides controlling plant growth and development under normal conditions, also mediate plant responses to salt stress (Yu et al., 2020). In this way, plants develop adaptive responses to salt stress by orchestrating the synthesis, signaling and metabolism of various hormones with multiple levels of crosstalk. ABA is one of the most important hormones in salt stress response that integrates numerous processes to cope with Na-induced osmotic stress and adaptive signaling cascades. Salinity and osmotic stress cause an increase in endogenous ABA levels for the activation of phosphorylation signaling cascades and ABA-responsive TFs belonging to different TF families. Accumulating evidence also uncovers the role of brassinosteroids in plant adaptation to salt stress, including genes associated to brassinosteroid synthesis and signaling (Yu et al., 2020). Brassinosteroids are perceived by cell surface-localized receptor kinases, BRI1 and BAK1 and trigger a series of phosphorylation/dephosphorylation events for the activation of brassinosteroid-responsive genes through the regulation of distinct TFs (BZR1, BES1) (Mao and Li, 2020; Yu et al., 2020). Besides, brassinosteroid signaling on plasma membrane has been shown to be important in salinity tolerance in rice (Dong et al., 2020). Thus, the presence of genes involved in brassinosteroid signaling in *indica* regions introgressed in IL22 (e.g., BAK1, BIP, and BSK) might well contribute to salinity tolerance in these plants (Figure 8). Brassinosteroid signaling also regulate salt tolerance by interacting with other hormone signaling pathways, such as ABA or ethylene pathways (Zhang et al., 2009; Divi et al., 2010; Li et al., 2012).

## Conclusion

To conclude, the results of this study indicated important transcriptional reprogramming caused by *indica* introgressions in IL22 plants which might well underlie the phenotype of salinity tolerance at the seedling stage in IL22 plants. It will be now of interest to use the transcriptome approach for the fine mapping and expression analysis of additional salt tolerant ILs generated in this study. This will help in deciphering precise functional interactions between *indica* and *japonica* genes in ILs contributing to salinity tolerance in rice. A better understanding of these mechanisms will be the basis for the development of new salt-tolerant rice varieties.

## Data Availability Statement

The datasets presented in this study can be found in online repositories. The names of the repository/repositories and accession number(s) can be found below: Gene Expression Omnibus, GSE167342.

## Author Contributions

BS and MB conceived the research plans and wrote the manuscript. MB performed the most experiments, genotyping (PCR, KASPar), phenotyping for salinity tolerance, RNA-Seq analysis, and analyzed the data. XS and SN performed FL478 × OLESA crosses. JG-A, HM-C, and LC contributed with hydroponic experiments. HM-C contributed to the validation of RNA-Seq data by RT-qPCR. JF, BC, and CG performed the GBS and analyzed the data. MP and GS performed Na^+^/K^+^ analyses and analyzed the data. All authors supervised and complemented the writing. BS agreed to serve as the author responsible for contact and ensures communication.

## Conflict of Interest

The authors declare that the research was conducted in the absence of any commercial or financial relationships that could be construed as a potential conflict of interest. The handling editor declared a past co-authorship with one of the author, GS.

## Publisher’s Note

All claims expressed in this article are solely those of the authors and do not necessarily represent those of their affiliated organizations, or those of the publisher, the editors and the reviewers. Any product that may be evaluated in this article, or claim that may be made by its manufacturer, is not guaranteed or endorsed by the publisher.

## Footnotes

1
https://www.ncbi.nlm.nih.gov/tools/primer-blast/

## Abbreviations

ABA: abscisic acid
DEG: differentially expressed gene
GBS: genotyping-by-sequencing
GO: Gene Ontology
HKT: high-affinity K^+^ transporter
IL: introgression line
KASPar: Kompetitive allele specific PCR
MABC: marker-assisted backcross
QTL: quantitative trait *loci*
ROS: reactive oxygen species
SES: Standard Evaluation System
SNP: single nucleotide polymorphism.

## References

[B1] Afzal Z. Howton T. C. Sun Y. Mukhtar M. S. (2016). The roles of aquaporins in plant stress responses. *J. Dev. Biol.* 4:9. 10.3390/jdb4010009 29615577 PMC5831814

[B2] Alam R. Sazzadur Rahman M. Seraj Z. I. Thomson M. J. Ismail A. M. Tumimbang-Raiz E. (2011). Investigation of seedling-stage salinity tolerance QTLs using backcross lines derived from *Oryza sativa* L. Pokkali. *Plant Breed.* 130 430–437. 10.1111/j.1439-0523.2010.01837.x

[B3] Asano T. Hakata M. Nakamura H. Aoki N. Komatsu S. Ichikawa H. (2011). Functional characterisation of OsCPK21, a calcium-dependent protein kinase that confers salt tolerance in rice. *Plant Mol. Biol.* 75 179–191. 10.1007/s11103-010-9717-1 21136139

[B4] Asano T. Hayashi N. Kobayashi M. Aoki N. Miyao A. Mitsuhara I. (2012). A rice calcium-dependent protein kinase OsCPK12 oppositely modulates salt-stress tolerance and blast disease resistance. *Plant J.* 69 26–36. 10.1111/j.1365-313X.2011.04766.x 21883553

[B5] Babu N. N. Krishnan S. G. Vinod K. K. Krishnamurthy S. L. Singh V. K. Singh M. P. (2017). Marker aided incorporation of saltol, a major QTL associated with seedling stage salt tolerance, into *Oryza sativa* “Pusa Basmati 1121”. *Front. Plant Sci.* 8:41. 10.3389/fpls.2017.00041 28184228 PMC5266695

[B6] Bajji M. Kinet J.-M. Lutts S. (2002). The use of the electrolyte leakage method for assessing cell membrane stability as a water stress tolerance test in durum wheat. *Plant Growth Regul.* 36 61–70. 10.1023/A:1014732714549

[B7] Batayeva D. Labaco B. Ye C. Li X. Usenbekov B. Rysbekova A. (2018). Genome-wide association study of seedling stage salinity tolerance in temperate japonica rice germplasm. *BMC Genet.* 19:2. 10.1186/s12863-017-0590-7 29298667 PMC5753436

[B8] Bonilla P. Dvorak J. Mackill D. Deal K. Gregorio G. (2002). RFLP and SSLP mapping of salinity tolerance genes in chromosome 1 of rice (*Oryza sativa* L.) using recombinant inbred lines. *Philipp. Agric. Sci.* 65 68–76.

[B9] Campo S. Baldrich P. Messeguer J. Lalanne E. Coca M. San Segundo B. (2014). Overexpression of a calcium-dependent protein kinase confers salt and drought tolerance in rice by preventing membrane lipid peroxidation. *Plant Physiol.* 165 688–704. 10.1104/pp.113.230268 24784760 PMC4044838

[B10] Chen H. C. Cheng W. H. Hong C. Y. Chang Y. S. Chang M. C. (2018). The transcription factor OsbHLH035 mediates seed germination and enables seedling recovery from salt stress through ABA-dependent and ABA-independent pathways, respectively. *Rice* 11:50. 10.1186/s12284-018-0244-z 30203325 PMC6134479

[B11] Cotsaftis O. Plett D. Johnson A. A. T. Walia H. Wilson C. Ismail A. M. (2011). Root-specific transcript profiling of contrasting rice genotypes in response to salinity stress. *Mol. Plant.* 4 25–41. 10.1093/MP/SSQ056 20924028

[B12] De Leon T. B. Linscombe S. Subudhi P. K. (2017). Identification and validation of QTLs for seedling salinity tolerance in introgression lines of a salt tolerant rice landrace “Pokkali”. *PLoS One* 12:e0175361. 10.1371/journal.pone.0175361 28388633 PMC5384751

[B13] Demidchik V. Straltsova D. Medvedev S. S. Pozhvanov G. A. Sokolik A. Yurin V. (2014). Stress-induced electrolyte leakage: the role of K+-permeable channels and involvement in programmed cell death and metabolic adjustment. *J. Exp. Bot.* 65 1259–1270. 10.1093/jxb/eru004 24520019

[B14] Divi U. K. Rahman T. Krishna P. (2010). Brassinosteroid-mediated stress tolerance in *Arabidopsis* shows interactions with abscisic acid, ethylene and salicylic acid pathways. *BMC Plant Biol.* 10:151. 10.1186/1471-2229-10-151 20642851 PMC3095295

[B15] Dong N. Yin W. Liu D. Zhang X. Yu Z. Huang W. (2020). Regulation of brassinosteroid signaling and salt resistance by SERK2 and potential utilization for crop improvement in rice. *Front Plant Sci.* 11:621859. 10.3389/fpls.2020.621859 33362843 PMC7758213

[B16] Dubouzet J. G. Sakuma Y. Ito Y. Kasuga M. Dubouzet E. G. Miura S. (2003). OsDREB genes in rice, *Oryza sativa* L., encode transcription activators that function in drought-, high-salt- and cold-responsive gene expression. *Plant J.* 33 751–763. 10.1046/j.1365-313x.2003.01661.x 12609047

[B17] Frouin J. Languillaume A. Mas J. Mieulet D. Boisnard A. Labeyrie A. (2018). Tolerance to mild salinity stress in japonica rice: a genome-wide association mapping study highlights calcium signaling and metabolism genes. *PLoS One* 13:e0190964. 10.1371/journal.pone.0190964 29342194 PMC5771603

[B18] Gregorio G. (1997). *Tagging Salinity Tolerance Genes in Rice Using Amplified Fragment Length Polymorphism (AFLP).* Rome: FAO.

[B19] Gregorio G. B. Senadhira D. (1993). Genetic analysis of salinity tolerance in rice (*Oryza sativa* L.). *Theor. Appl. Genet.* 86 333–338. 10.1007/BF00222098 24193479

[B20] Gregorio G. B. Senadhira D. Mendoza R. D. (1997). *Screening Rice for Salinity Tolerance.* Laguna: International Rice Research Institute.

[B21] Gregorio G. B. Senadhira D. Mendoza R. D. Manigbas N. L. Roxas J. P. Guerta C. Q. (2002). Progress in breeding for salinity tolerance and associated abiotic stresses in rice. *Field Crop Res.* 76 91–101. 10.1016/S0378-4290(02)00031-X

[B22] Gui J. Zheng S. Liu C. Shen J. Li J. Li L. (2016). OsREM4.1 interacts with OsSERK1 to coordinate the interlinking between abscisic acid and brassinosteroid signaling in rice. *Dev. Cell.* 38 201–213. 10.1016/j.devcel.2016.06.011 27424498

[B23] Guo Q. Liu L. Barkla B. J. (2019). Membrane lipid remodeling in response to salinity. *Int. J. Mol. Sci.* 20:4264. 10.3390/ijms20174264 31480391 PMC6747501

[B24] Han J.-H. Shin N.-H. Moon J.-H. Yi C. Yoo S.-C. Chin J. H. (2020). Genetic and phenotypic characterization of rice backcrossed inbred sister lines of saltol in temperate saline reclaimed area. *Plant Breed. Biotechnol.* 8 58–86. 10.9787/pbb.2020.8.1.58

[B25] Hong Y. Zhang H. Huang L. Li D. Song F. (2016). Overexpression of a stress-responsive NAC transcription factor gene ONAC022 improves drought and salt tolerance in rice. *Front. Plant Sci.* 7:4. 10.3389/fpls.2016.00004 26834774 PMC4722120

[B26] Hossain M. S. Dietz K. J. (2016). Tuning of redox regulatory mechanisms, reactive oxygen species and redox homeostasis under salinity stress. *Front. Plant Sci.* 7:548. 10.3389/fpls.2016.00548 27242807 PMC4861717

[B27] Hu H. Dai M. Yao J. Xiao B. Li X. Zhang Q. (2006). Overexpressing a NAM, ATAF, and CUC (NAC) transcription factor enhances drought resistance and salt tolerance in rice. *Proc. Natl. Acad. Sci. U.S.A.* 103 12987–12992. 10.1073/pnas.0604882103 16924117 PMC1559740

[B28] Hu Y. Yu D. (2014). BRASSINOSTEROID INSENSITIVE2 interacts with ABSCISIC ACID INSENSITIVE5 to mediate the antagonism of brassinosteroids to abscisic acid during seed germination in *Arabidopsis*. *Plant Cell* 26 4394–4408. 10.1105/tpc.114.130849 25415975 PMC4277219

[B29] Hunter K. Kimura S. Rokka A. Tran H. C. Toyota M. Kukkonen J. P. (2019). CRK2 enhances salt tolerance by regulating callose deposition in connection with PLDα1. *Plant Physiol.* 180 2004–2021. 10.1104/pp.19.00560 31118265 PMC6670071

[B30] Hussain Q. Asim M. Zhang R. Khan R. Farooq S. Wu J. (2021). Transcription factors interact with ABA through gene expression and signaling pathways to mitigate drought and salinity stress. *Biomolecules* 11:1159. 10.3390/biom11081159 34439825 PMC8393639

[B31] Kaur N. Kirat K. Saini S. Sharma I. Gantet P. Pati P. K. (2016). Reactive oxygen species generating system and brassinosteroids are linked to salt stress adaptation mechanisms in rice. *Plant Signal. Behav.* 11:e1247136. 10.1080/15592324.2016.1247136 27739914 PMC5225940

[B32] Ketehouli T. Carther K. F. I. Noman M. Wang F. W. Li X. W. Li H. Y. (2019). Adaptation of plants to salt stress: characterization of Na+ and K+ transporters and role of Cbl gene family in regulating salt stress response. *Agronomy* 9:687. 10.3390/agronomy9110687

[B33] Kim S.-H. Bhat P. R. Cui X. Walia H. Xu J. Wanamaker S. (2009). Detection and validation of single feature polymorphisms using RNA expression data from a rice genome array. *BMC Plant Biol.* 9:65. 10.1186/1471-2229-9-65 19480680 PMC2697985

[B34] Kobayashi M. Ohura I. Kawakita K. Yokota N. Fujiwara M. Shimamoto K. (2007). Calcium-dependent protein kinases regulate the production of reactive oxygen species by potato NADPH oxidase. *Plant Cell* 19 1065–1080. 10.1105/tpc.106.048884 17400895 PMC1867354

[B35] Kobayashi N. I. Yamaji N. Yamamoto H. Okubo K. Ueno H. Costa A. (2017). OsHKT1;5 mediates Na+ exclusion in the vasculature to protect leaf blades and reproductive tissues from salt toxicity in rice. *Plant J.* 91 657–670. 10.1111/tpj.13595 28488420

[B36] Kurowska M. M. (2021). Aquaporins in cereals—important players in maintaining cell homeostasis under abiotic stress. *Genes* 12:477. 10.3390/genes12040477 33806192 PMC8066221

[B37] Li G.-W. Peng Y.-H. Yu X. Zhang M.-H. Cai W.-M. Sun W.-N. (2008). Transport functions and expression analysis of vacuolar membrane aquaporins in response to various stresses in rice. *J. Plant Physiol.* 165 1879–1888. 10.1016/j.jplph.2008.05.002 18707797

[B38] Li Y.-F. Zheng Y. Vemireddy L. R. Panda S. K. Jose S. Ranjan A. (2018). Comparative transcriptome and translatome analysis in contrasting rice genotypes reveals differential mRNA translation in salt-tolerant Pokkali under salt stress. *BMC Genomics* 19:935. 10.1186/s12864-018-5279-4 30598105 PMC6311934

[B39] Li Z. Y. Xu Z. S. He G. Y. Yang G. X. Chen M. Li L. C. (2012). A mutation in *Arabidopsis* BSK5 encoding a brassinosteroid-signaling kinase protein affects responses to salinity and abscisic acid. *Biochem. Biophys. Res. Commun.* 426 522–527. 10.1016/j.bbrc.2012.08.118 22982312

[B40] Liu K. Wang L. Xu Y. Chen N. Ma Q. Li F. (2007). Overexpression of OsCOIN, a putative cold inducible zinc finger protein, increased tolerance to chilling, salt and drought, and enhanced proline level in rice. *Planta* 226 1007–1016. 10.1007/s00425-007-0548-5 17549515

[B41] Liu Q. Umeda M. Uchimiya H. (1994). Isolation and expression analysis of two rice genes encoding the major intrinsic protein. *Plant Mol. Biol.* 26 2003–2007. 10.1007/BF00019511 7858235

[B42] López-Cristoffanini C. Bundó M. Serrat X. San Segundo B. López-Carbonell M. Nogués S. (2020). A comprehensive study of the proteins involved in salinity stress response in roots and shoots of the FL478 genotype of rice (*Oryza sativa* L. ssp. indica). *Crop J.* 9 1154–1168. 10.1016/j.cj.2020.10.009

[B43] Mallikarjuna G. Mallikarjuna K. Reddy M. K. Kaul T. (2011). Expression of OsDREB2A transcription factor confers enhanced dehydration and salt stress tolerance in rice (*Oryza sativa* L.). *Biotech. Lett.* 33 1689–1697. 10.1007/s10529-011-0620-x 21528404

[B44] Mao J. Li J. (2020). Regulation of three key kinases of brassinosteroid signaling pathway. *Int. J. Mol. Sci.* 21:4340. 10.3390/ijms21124340 32570783 PMC7352359

[B45] Martinez-Medina A. Flors V. Heil M. Mauch-Mani B. Pieterse C. M. J. Pozo M. J. (2016). Recognizing plant defense priming. *Trends Plant Sci.* 21 818–822. 10.1016/j.tplants.2016.07.009 27507609

[B46] Nakashima K. Tran L. S. Van Nguyen D. Fujita M. Maruyama K. Todaka D. (2007). Functional analysis of a NAC-type transcription factor OsNAC6 involved in abiotic and biotic stress-responsive gene expression in rice. *Plant J.* 51 617–630. 10.1111/j.1365-313X.2007.03168.x 17587305

[B47] Nakashima K. Yamaguchi-Shinozaki K. Shinozaki K. (2014). The transcriptional regulatory network in the drought response and its crosstalk in abiotic stress responses including drought, cold, and heat. *Front. Plant Sci.* 5:170. 10.3389/fpls.2014.00170 24904597 PMC4032904

[B48] Negrão S. Courtois B. Ahmadi N. Abreu I. Saibo N. Oliveira M. M. (2011). Recent updates on salinity stress in rice: from physiological to molecular responses. *CRC Crit. Rev. Plant Sci.* 30 329–377. 10.1080/07352689.2011.587725

[B49] Nutan K. K. Kushwaha H. R. Singla-Pareek S. L. Pareek A. (2017). Transcription dynamics of Saltol QTL localized genes encoding transcription factors, reveals their differential regulation in contrasting genotypes of rice. *Funct. Integr. Genomics.* 17 69–83. 10.1007/s10142-016-0529-5 27848097

[B50] Nutan K. K. Singla-Pareek S. L. Pareek A. (2020). The Saltol QTL-localized transcription factor OsGATA8 plays an important role in stress tolerance and seed development in *Arabidopsis* and rice. *J. Exp. Bot.* 71 684–698. 10.1093/jxb/erz368 31613368

[B51] Ohnishi T. Yoshino M. Yamakawa H. Kinoshita T. (2011). The biotron breeding system: a rapid and reliable procedure for genetic studies and breeding in rice. *Plant Cell Physiol.* 52 1249–1257. 10.1093/pcp/pcr066 21622665

[B52] Platten J. D. Egdane J. A. Ismail A. M. (2013). Salinity tolerance, Na+ exclusion and allele mining of HKT1;5 in Oryza sativa and O. glaberrima: many sources, many genes, one mechanism? *BMC Plant Biol.* 13:32. 10.1186/1471-2229-13-32 23445750 PMC3599985

[B53] Ren Z. H. Gao J. P. Li L. G. Cai X. L. Huang W. Chao D. Y. (2005). A rice quantitative trait locus for salt tolerance encodes a sodium transporter. *Nat. Genet.* 37 1141–1146. 10.1038/ng1643 16155566

[B54] Shabala S. Alnayef M. Bose J. Chen Z.-H. Venkataraman G. Zhou M. (2021). Revealing the role of the calcineurin B-like protein-interacting protein kinase 9 (CIPK9) in rice adaptive responses to salinity, osmotic stress, and K+ deficiency. *Plants* 10:1513. 10.3390/plants10081513 34451561 PMC8399971

[B55] Shah W. H. Rasool A. Saleem S. Mushtaq N. U. Tahir I. Hakeem K. R. (2021). Understanding the Integrated Pathways and mechanisms of transporters, protein kinases, and transcription factors in plants under salt stress. *Int. J. Genomics* 2021:5578727. 10.1155/2021/5578727 33954166 PMC8057909

[B56] Sharma I. Ching E. Saini S. Bhardwaj R. Pati P. K. (2013). Exogenous application of brassinosteroid offers tolerance to salinity by altering stress responses in rice variety Pusa Basmati-1. *Plant Physiol. Biochem.* 69 17–26. 10.1016/j.plaphy.2013.04.013 23707881

[B57] Sharma I. Kaur N. Pati P. K. (2017). Brassinosteroids: a promising option in deciphering remedial strategies for abiotic stress tolerance in rice. *Front. Plant Sci.* 8:2151. 10.3389/fpls.2017.02151 29326745 PMC5742319

[B58] Soda N. Kushwaha H. R. Soni P. Singla-Pareek S. L. Pareek A. (2013). A suite of new genes defining salinity stress tolerance in seedlings of contrasting rice genotypes. *Funct. Integr. Genomics* 13 351–365. 10.1007/s10142-013-0328-1 23813016

[B59] Supek F. Bošnjak M. Škunca N. Šmuc T. (2011). REVIGO summarizes and visualizes long lists of gene ontology terms. *PLoS One* 6:e21800. 10.1371/journal.pone.0021800 21789182 PMC3138752

[B60] Tan T. Cai J. Zhan E. Yang Y. Zhao J. Guo Y. (2016). Stability and localization of 14-3-3 proteins are involved in salt tolerance in *Arabidopsis*. *Plant Mol. Biol.* 92 391–400. 10.1007/s11103-016-0520-5 27503471

[B61] Thomson M. J. de Ocampo M. Egdane J. Rahman M. A. Sajise A. G. Adorada D. L. (2010). Characterizing the Saltol quantitative trait locus for salinity tolerance in rice. *Rice* 3 148–160. 10.1007/s12284-010-9053-8

[B62] Tian T. Liu Y. Yan H. You Q. Yi X. Du Z. (2017). AgriGO v2.0: a GO analysis toolkit for the agricultural community, 2017 update. *Nucleic Acids Res.* 3 122–129. 10.1093/nar/gkx382 28472432 PMC5793732

[B63] Tian Y. Fan M. Qin Z. Lv H. Wang M. Zhang Z. (2018). Hydrogen peroxide positively regulates brassinosteroid signaling through oxidation of the BRASSINAZOLE-RESISTANT1 transcription factor. *Nat. Commun.* 9:1963. 10.1038/s41467-018-03463-x 29540799 PMC5852159

[B64] Usatov A. V. Alabushev A. V. Kostylev P. I. Azarin K. V. Makarenko M. S. Usatova O. A. (2015). Introgression the SalTol QTL into the elite rice variety of Russia by marker-assisted selection. *Am. J. Agric. Biol. Sci.* 10 165–169. 10.3844/ajabssp.2015.165.169

[B65] Van Zelm E. Zhang Y. Testerink C. (2020). Salt tolerance mechanisms of plants. *Annu. Rev. Plant Biol.* 71 403–433. 10.1146/annurev-arplant-050718-100005 32167791

[B66] Walia H. Wilson C. Condamine P. Liu X. Ismail A. M. Zeng L. (2005). Comparative transcriptional profiling of two contrasting rice genotypes under salinity stress during the vegetative growth stage. *Plant Physiol.* 139 822–835. 10.1104/pp.105.065961 16183841 PMC1255998

[B67] Wang J. Nan N. Li N. Liu Y. Wang T. J. Hwang I. (2020). A DNA methylation reader-chaperone regulator-transcription factor complex activates OsHKT1;5 expression during salinity stress. *Plant Cell* 32 3535–3558. 10.1105/tpc.20.00301 32938753 PMC7610284

[B68] Wang Q. Guan Y. Wu Y. Chen H. Chen F. Chu C. (2008). Overexpression of a rice OsDREB1F gene increases salt, drought, and low temperature tolerance in both *Arabidopsis* and rice. *Plant Mol. Biol.* 67 589–602. 10.1007/s11103-008-9340-6 18470484

[B69] Wang Y. Blatt M. R. Chen Z.-H. (2018). Ion transport at the plant plasma membrane. *eLS* 1:a0001307.

[B70] Waziri A. Kumar P. Purty R. S. (2016). Saltol QTL and their role in salinity tolerance in rice. *Austin J. Biotechnol. Bioeng.* 3 1067–1072.

[B71] Xiang Y. Tang N. Du H. Ye H. Xiong L. (2008). Characterization of OsbZIP23 as a key player of the basic leucine zipper transcription factor family for conferring abscisic acid sensitivity and salinity and drought tolerance in rice. *Plant Physiol.* 148 1938–1952. 10.1104/pp.108.128199 18931143 PMC2593664

[B72] Yadav A. K. Kumar A. Grover N. Ellur R. K. Krishnan S. G. Bollinedi H. (2020). Marker aided introgression of ‘Saltol’, a major QTL for seedling stage salinity tolerance into an elite Basmati rice variety ‘Pusa Basmati 1509’. *Sci. Rep.* 10:13877. 10.1038/s41598-020-70664-0 32887905 PMC7474085

[B73] Yang Y. Guo Y. (2018). Unraveling salt stress signaling in plants. *J. Integr. Plant Biol.* 60 796–804. 10.1111/jipb.12689 29905393

[B74] Yoon Y. Seo D. H. Shin H. Kim H. J. Kim C. M. Jang G. (2020). The role of stress-responsive transcription factors in modulating abiotic stress tolerance in plants. *Agronomy* 10:788. 10.3390/agronomy10060788

[B75] Yoshida T. Mogami J. Yamaguchi-Shinozaki K. (2014). ABA-dependent and ABA-independent signaling in response to osmotic stress in plants. *Curr. Opin. Plant Biol.* 21 133–139. 10.1016/j.pbi.2014.07.009 25104049

[B76] Yu Z. Duan X. Luo L. Dai S. Ding Z. Xia G. (2020). How plant hormones mediate salt stress responses. *Trends Plant Sci.* 25 1117–1130. 10.1016/j.tplants.2020.06.008 32675014

[B77] Zagorchev L. Kamenova P. Odjakova M. (2014). The role of plant cell wall proteins in response to salt stress. *Sci. World J.* 19 764089. 10.1155/2014/764089 24574917 PMC3916024

[B78] Zeng L. Shannon M. C. (2000). Salinity effects on seedling growth and yield components of rice. *Crop Sci.* 40 996–1003. 10.2135/cropsci2000.404996x 34798789

[B79] Zhang S. Cai Z. Wang X. (2009). The primary signaling outputs of brassinosteroids are regulated by abscisic acid signaling. *Proc. Natl. Acad. Sci. U.S.A.* 106 4543–4548. 10.1073/pnas.0900349106 19240210 PMC2657416

[B80] Zhou J. Wang J. Li X. Xia X. J. Zhou Y. H. Shi K. (2014). H2O2 mediates the crosstalk of brassinosteroid and abscisic acid in tomato responses to heat and oxidative stresses. *J. Exp. Bot.* 65 4371–4383. 10.1093/jxb/eru217 24899077 PMC4112640

[B81] Zhu X. Pan T. Zhang X. Fan L. Quintero F. J. Zhao H. (2018). K(+) efflux antiporters 4, 5, and 6 mediate pH and K(+) homeostasis in endomembrane compartments. *Plant Physiol.* 178 1657–1678. 10.1104/pp.18.01053 30309966 PMC6288736

