# Supplementary material for: Integrative Approach for Precise Genotyping and Transcriptomics of Salt Tolerant Introgression Rice Lines
Source: Front Plant Sci. 2022 Jan 21;12:797141. doi: 10.3389/fpls.2021.797141 (PMC8813771; doi:10.3389/fpls.2021.797141)
Supplement: Supplementary file 1 [file Data_Sheet_1.PDF]

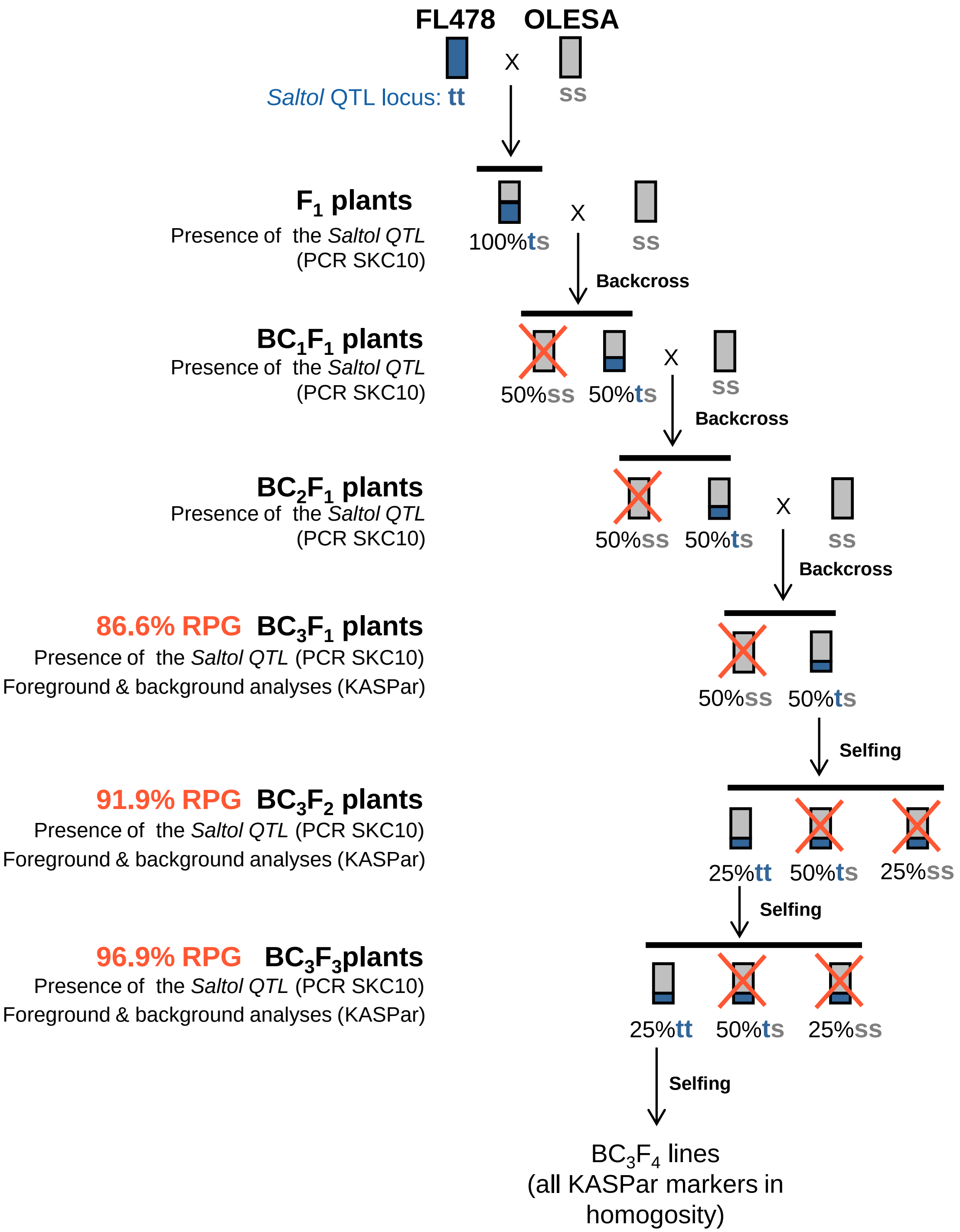

Supplementary Figure 1. Breeding scheme used in the marked-assisted backcross introgression of the *Saltol* QTL from FL478 (*indica*) into the background of the rice variety OLESA (temperate *japonica* rice).

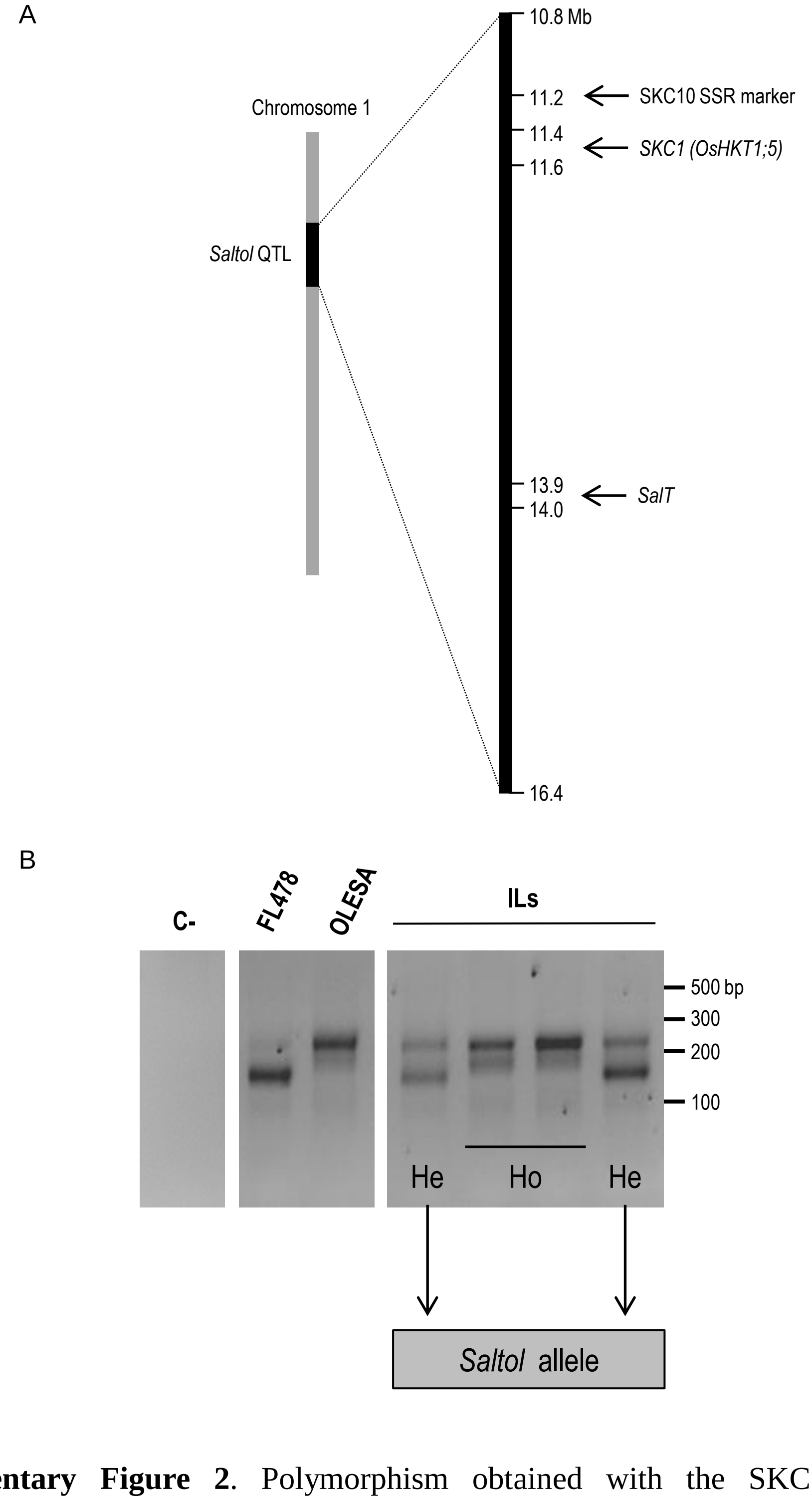

Supplementary Figure 2. Polymorphism obtained with the SKC10 SSR marker visualized by agarose gel analysis. **(A)** *Saltol* QTL region showing the SKC10 SSR marker and relevant salt-related genes positions. **(B)** PCR products obtained from the *Saltol* donor (FL478), the recurrent (OLESA) parent and 4 representative introgression lines derived from FL478 x OLESA crosses (BC_2F_1). C-, negative control, He, heterozygous, Ho, homozygous. Primers are indicated in Supplementary Table 2.

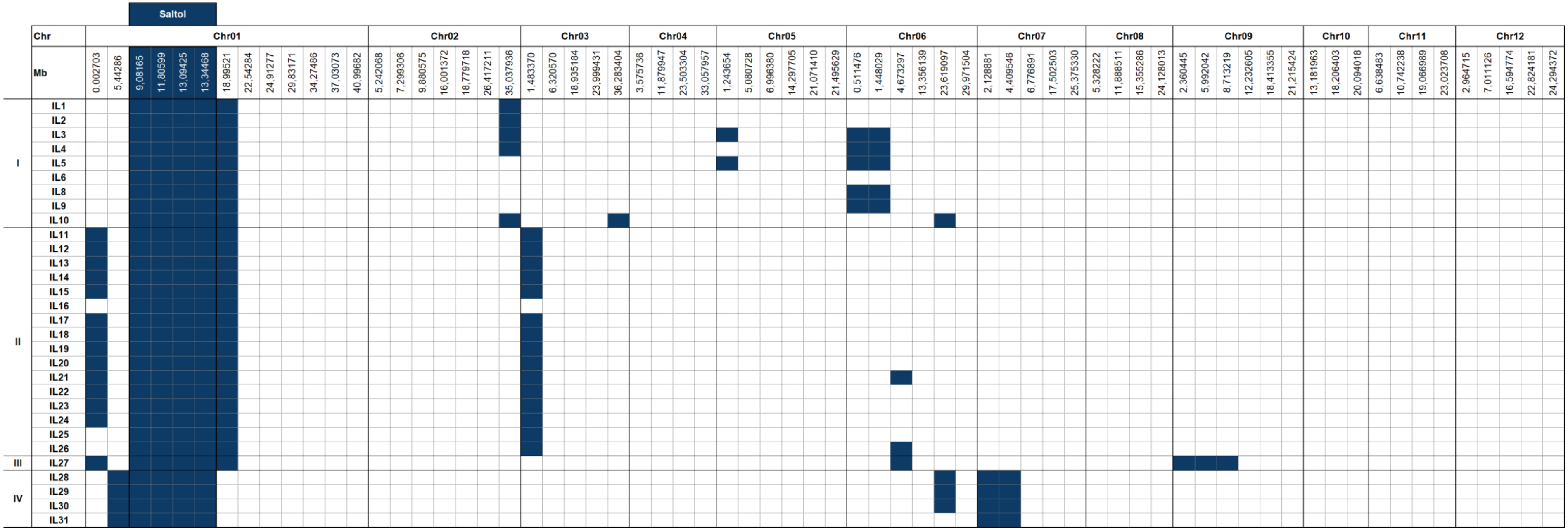

Supplementary Figure 3. Graphical representation of the genotypes of the *Saltol*-introgressed rice lines (BC_3F_3). Genotyping was carried out by KASPar analysis. SNPs are indicated in columns according to their chromosomal location (in mega base-pairs, Mb). Introgression lines (IL1 to IL31) are clustered in four groups (I to IV) depending on the BC_3F_1 parent from which they derive. The *Saltol* QTL location (and length) is indicated in the upper part. Homozygous donor (FL478) and recurrent (OLESA) alleles are depicted in blue and white, respectively. The KASPar markers used in this study are listed in Supplementary Tables 1 and 2.

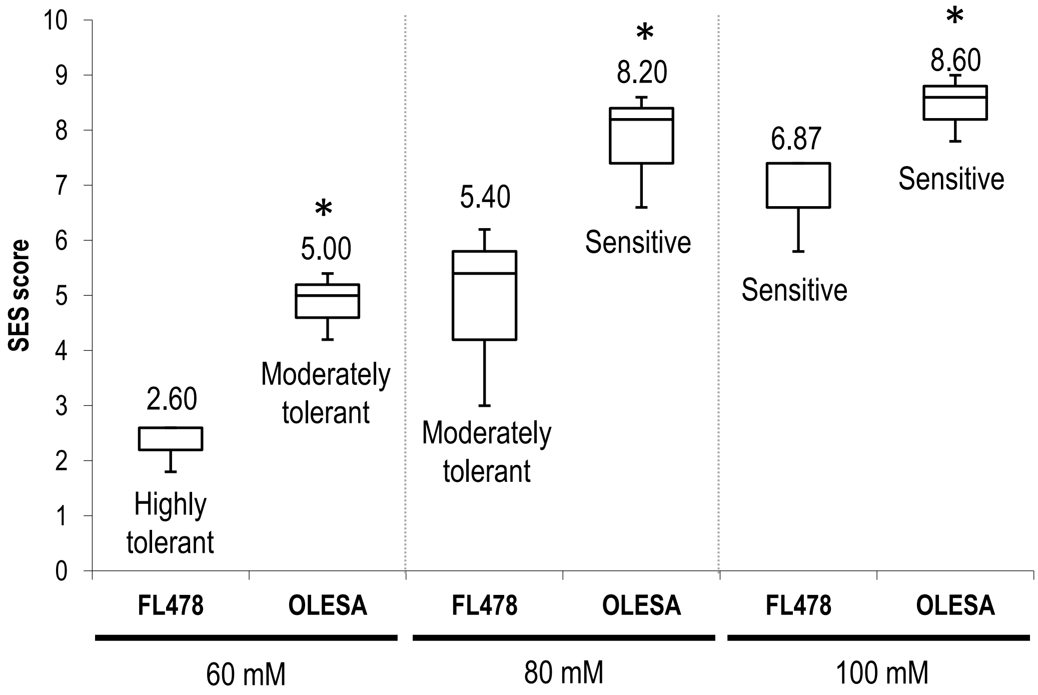

Supplementary Figure 4. SES score of parental lines (FL478, OLESA) hydroponically grown in modified Yoshida solution containing different NaCl concentrations (60 mM, 80 mM and 100 mM) for 14 days. Box plots show the distribution of SES scores in each line and condition (15 plants/genotype each experiment; T-test, * $P < 0.05$). Values above each box indicate the mean SES score .

A

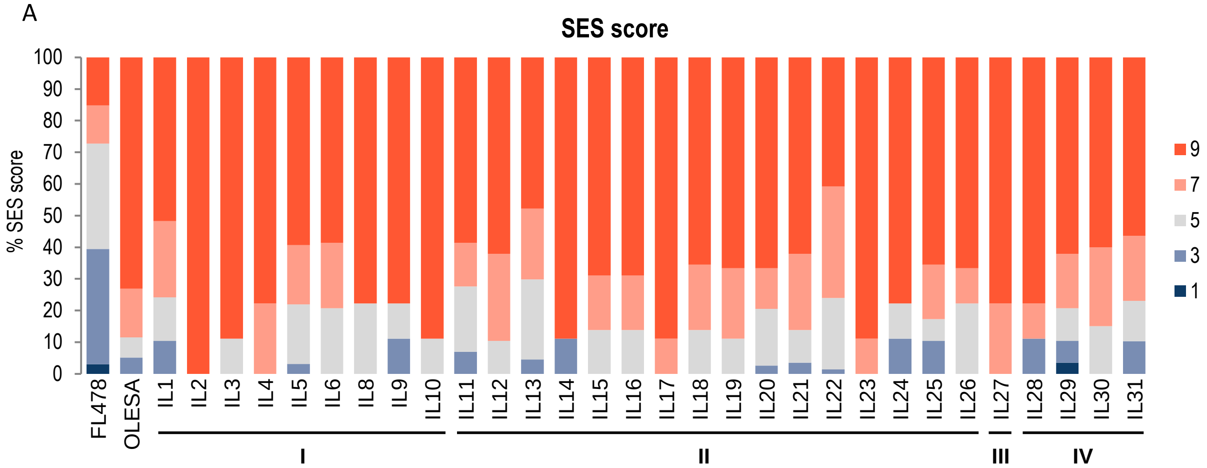

B

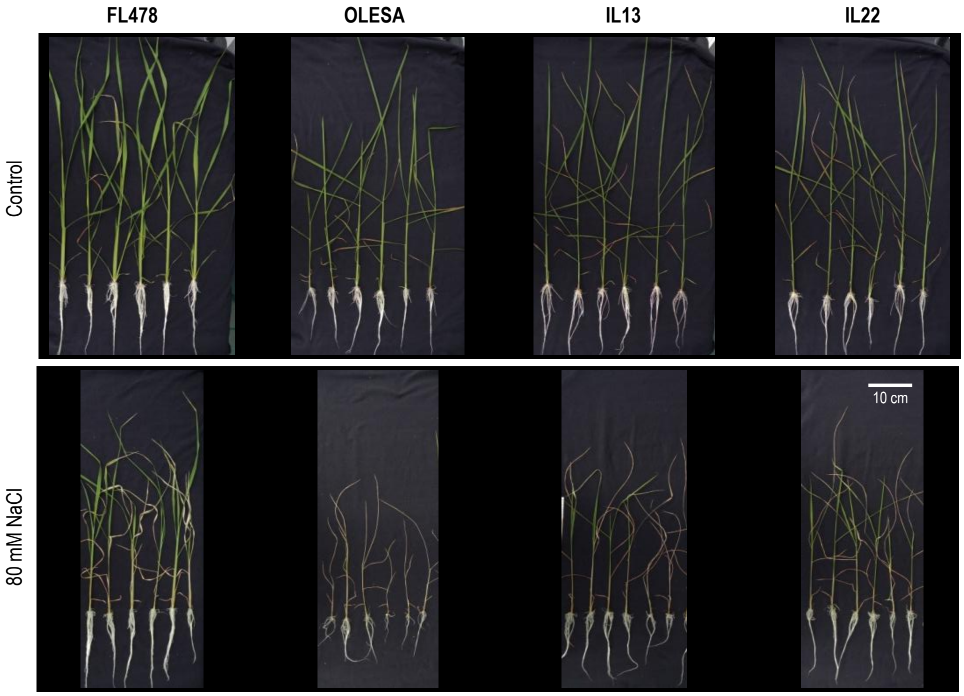

Supplementary Figure 5. Characterization of salt tolerant introgression lines. **(A)** Standard evaluation system (SES) scores of visual salt injury of the 30 ILs. Evaluation was performed after 14 days of salt treatment (80 mM NaCl). SES scores are shown as the percentage of plants at each score value. 1, highly tolerant; 3, tolerant; 5, moderately tolerant; 7, sensitive; 9, highly sensitive. ILs are clustered in four groups (I to IV) depending on the BC_3F_1 parent used. ILs were evaluated in successive rounds, with 5 plants and 10 plants in control and salt conditions respectively in each experiment, and most salt-sensitive ILs were discarded in the following assays. A total of six independent experiments were carried out with the most salt-tolerant ILs. **(B)** Representative images of IL22 and IL13 plants and parental lines in control and salt conditions after 14 days of treatment.

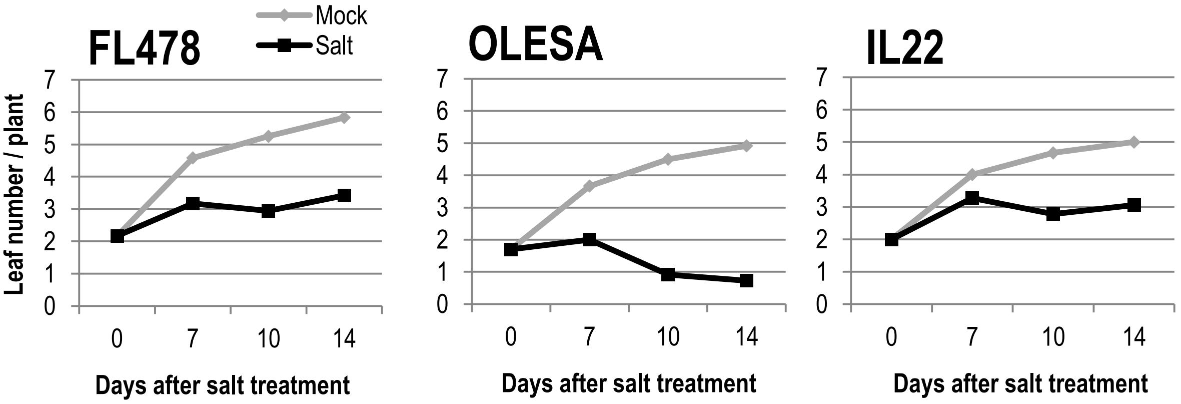

Supplementary Figure 6. Plant growth of parental lines (FL478, OLESA) and IL22 plants hydroponically grown in modified Yoshida solution containing 80 mM NaCl. Control plants were not supplemented with NaCl. The leaf number of each genotype at different times of salt treatment is indicated. At least 6 plants per genotype and condition were assayed.

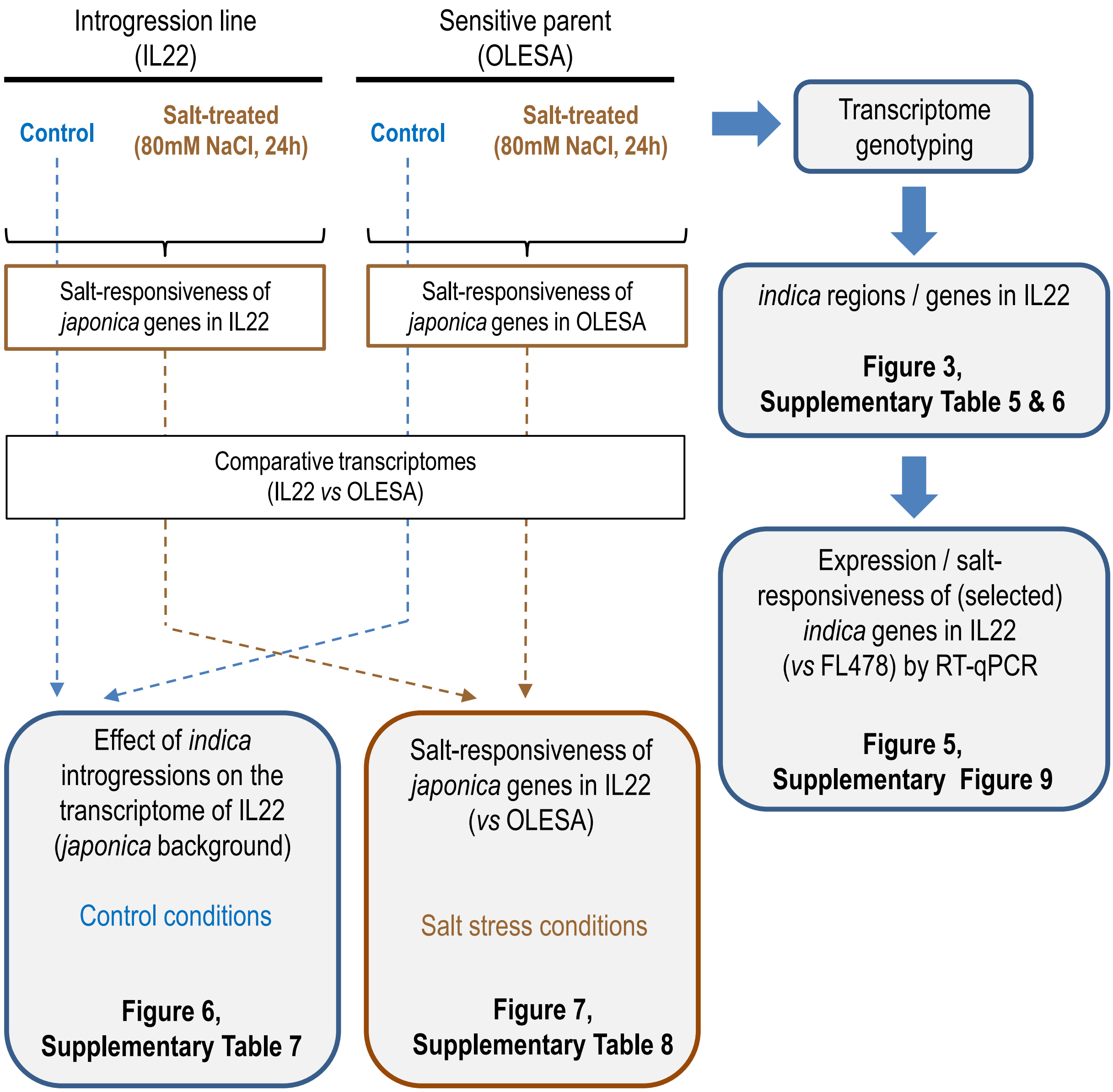

Supplementary Figure 7. Samples analysed by RNASeq, and comparisons of data sets from each genotype (IL22, OLESA) and condition (control, salt-treated).

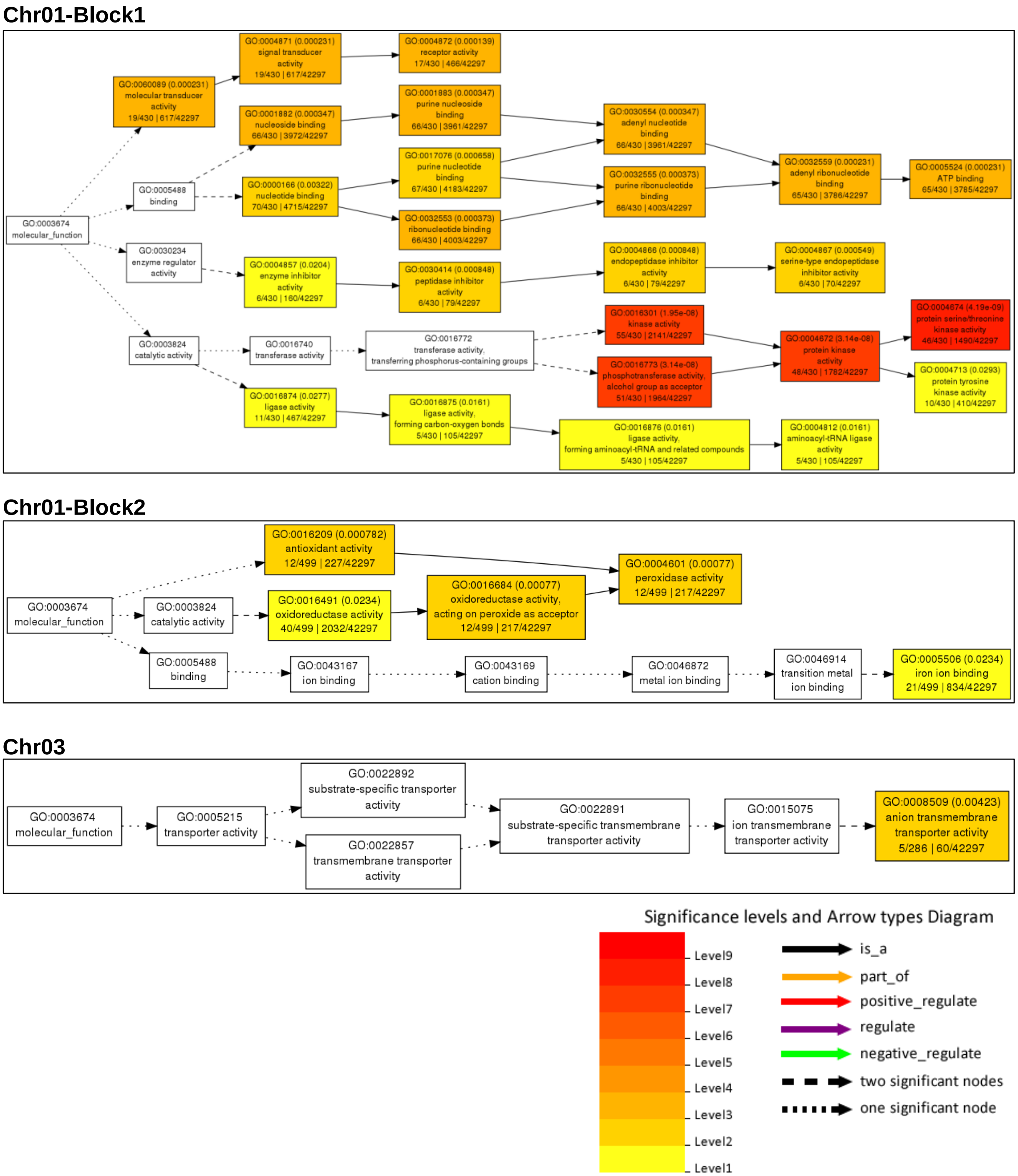

Supplementary Figure 8. Singular enrichment analysis of introgressed *indica* genes (chromosome 1, blocks 1 and 2, and chromosome 3) using AgriGO (Tian *et al.*, 2017). For a full list of gene IDs, see Supplementary Table 5.

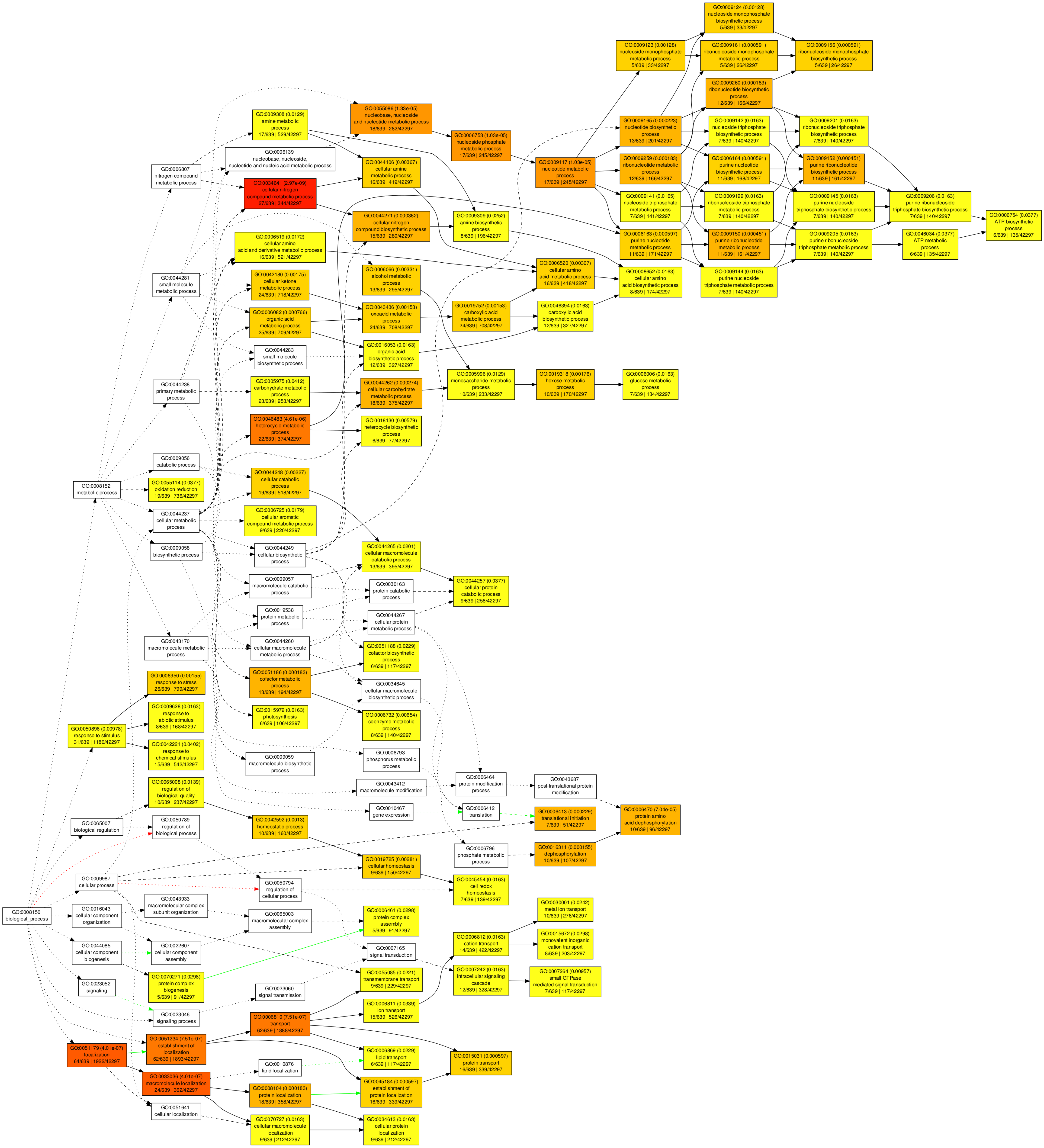

Supplementary Figure 9. Singular enrichment analysis of *japonica* genes up-regulated in IL22 plants at 24 h of salt treatment (80 mM NaCl) using AgriGO (Tian *et al.*, 2017). For a full list of gene IDs, see Supplementary Table 8.

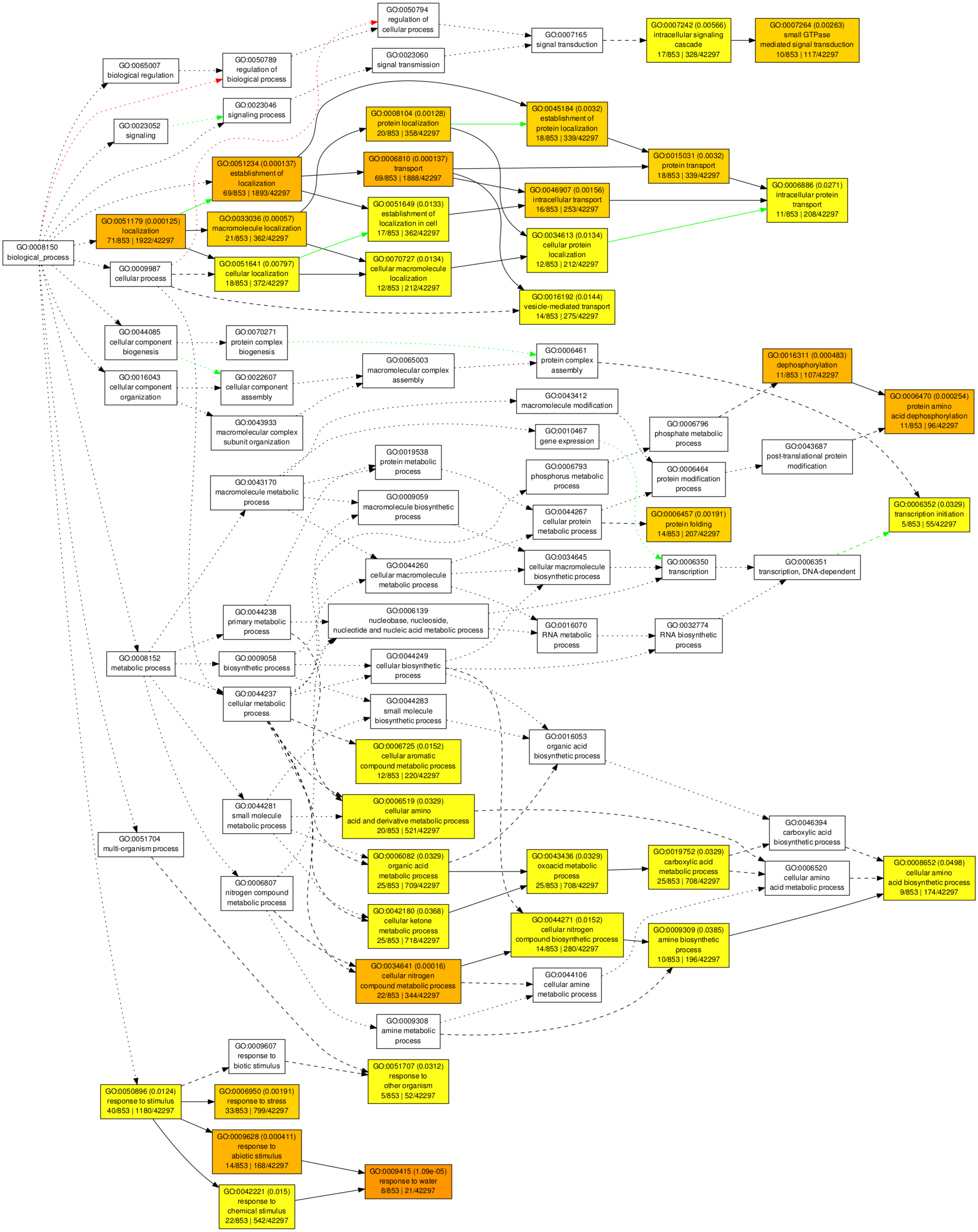

Supplementary Figure 10. Singular enrichment analysis of *japonica* genes up-regulated in OLESA plants at 24 h of salt treatment (80 mM NaCl) using AgriGO (Tian *et al.*, 2017). For a full list of gene IDs, see Supplementary Table 8.

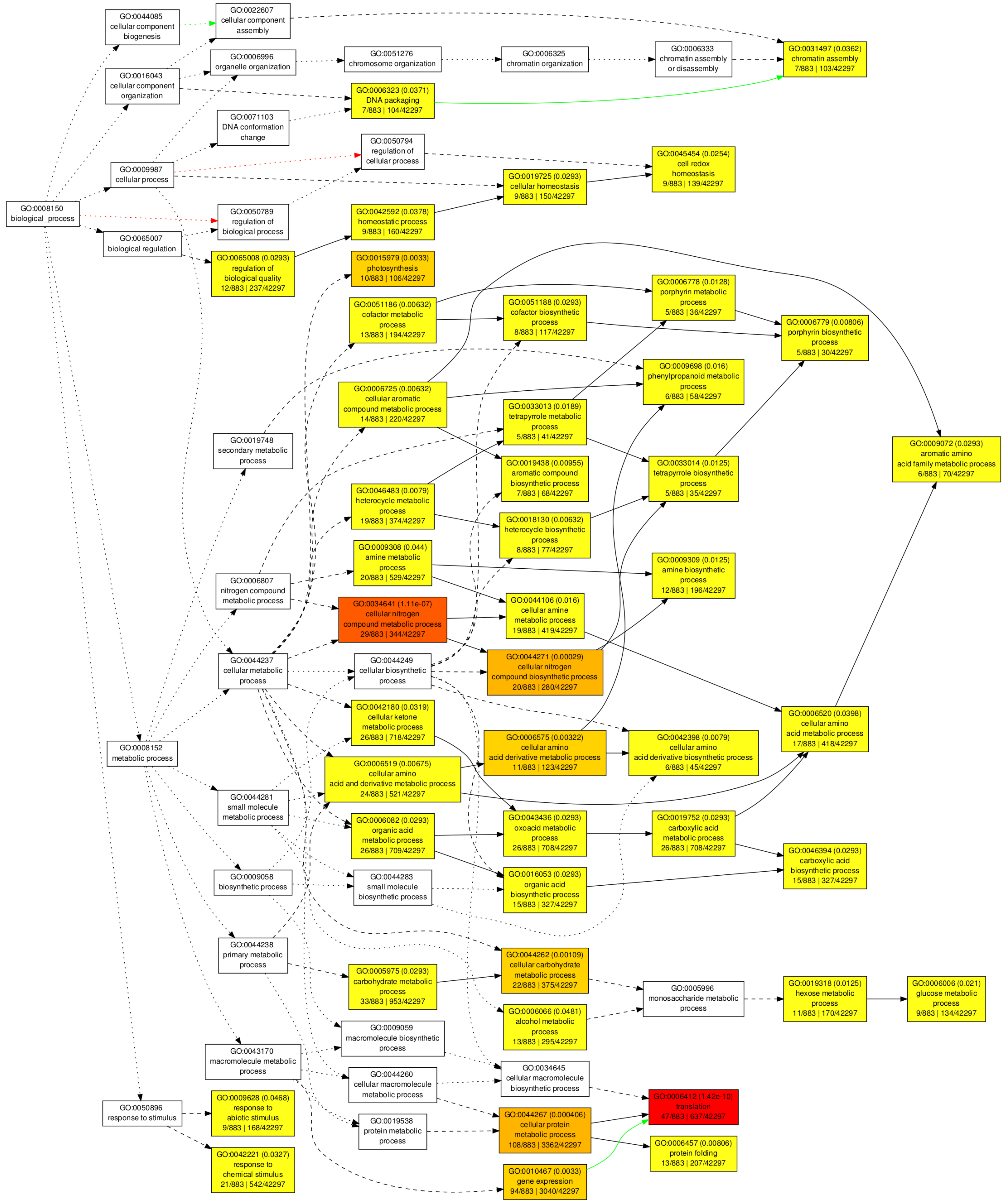

Supplementary Figure 11. Singular enrichment analysis of *japonica* genes down-regulated in IL22 plants at 24 h of salt treatment (80 mM NaCl) using AgriGO (Tian *et al.*, 2017). For a full list of gene IDs, see Supplementary Table 8.

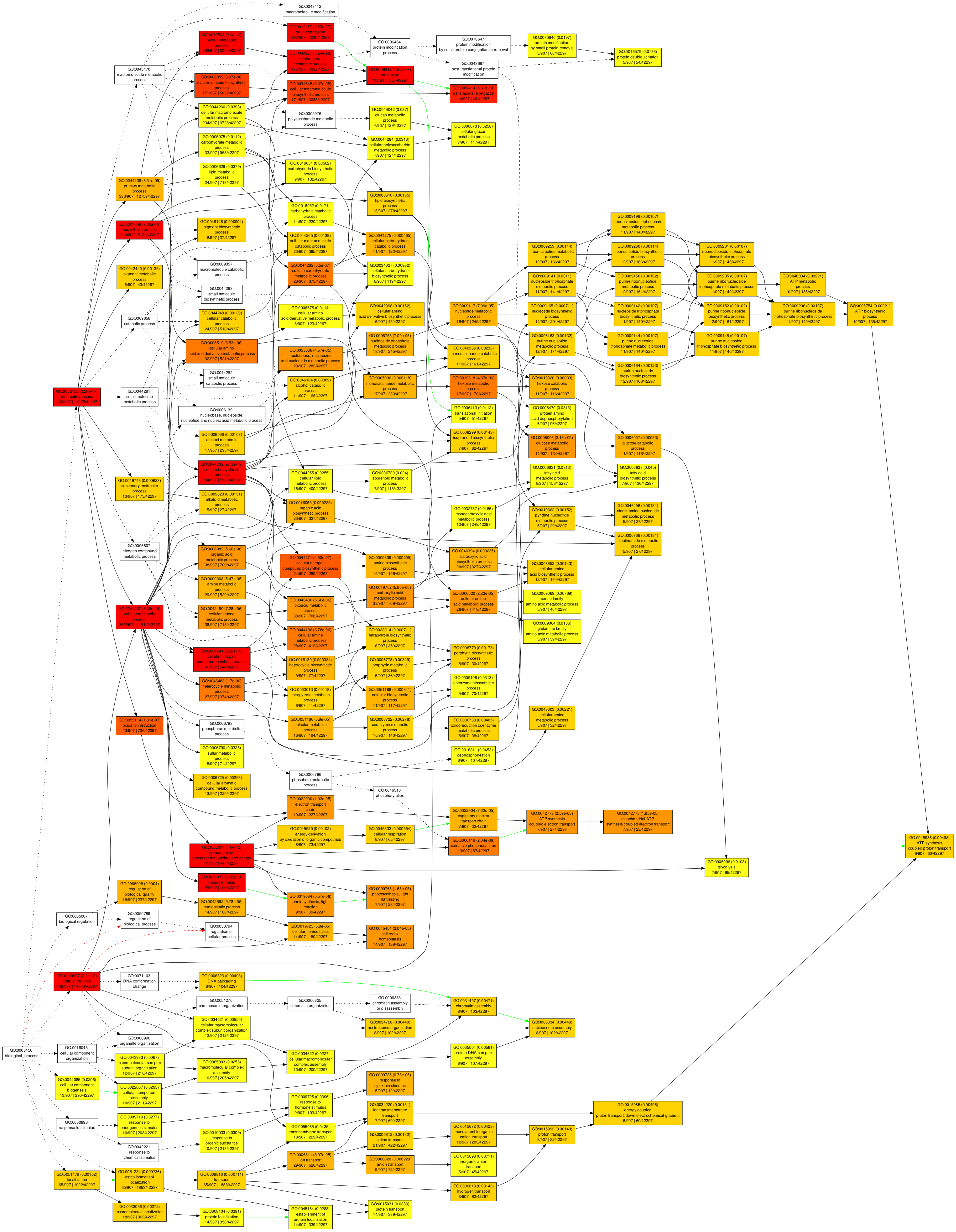

Supplementary Figure 12. Singular enrichment analysis of *japonica* genes down-regulated in OLESA plants at 24 h of salt treatment (80 mM NaCl) using AgriGO (Tian *et al.*, 2017). For a full list of gene IDs, see Supplementary Table 8.

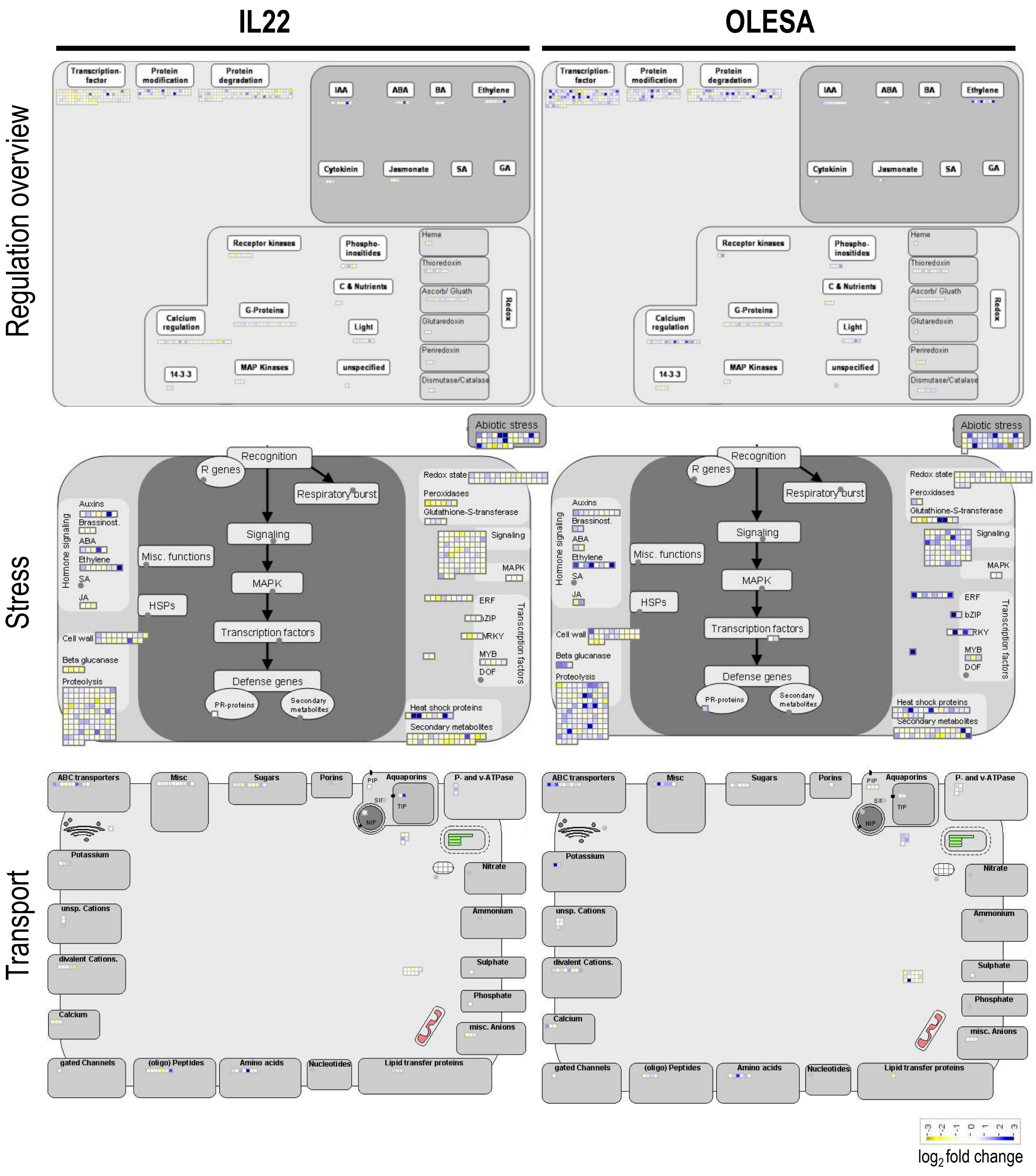

Supplementary Figure 13. Mapman analysis of *japonica* genes up- and down-regulated in IL22 and OLESA plants at 24 h of salt treatment (80 mM NaCl). Regulation overview, stress and transport schemes are shown. Color scale (yellow to blue) represents the $\log_2$ fold change of salt vs. control conditions.
