# Supplementary material for: Integrative Approach for Precise Genotyping and Transcriptomics of Salt Tolerant Introgression Rice Lines
Source: Front Plant Sci. 2022 Jan 21;12:797141. doi: 10.3389/fpls.2021.797141 (PMC8813771; doi:10.3389/fpls.2021.797141)
Supplement: Supplementary file 2 [file Data_Sheet_2.docx]

**Supplementary Methods**

*Transcriptome analysis by RNA-Seq and identification of introgressed indica regions*

Total RNA was extracted from leaves of rice plants that have been grown in hydroponic culture using the Maxwell RSC Plant RNA Kit (Promega). Three biological replicates for each genotype (IL22 and OLESA) and condition (control and salt-treated plants) were examined. Each biological replicate consisted of leaves from four individual plants. RNA samples were quantified and quality tested by Agilent 2100 Bioanalyzer RNA assay (Agilent technologies, Santa Clara, CA) and only samples with an RNA integrity number (RIN) ≥ 8 were used.

TruSeq Stranded mRNA kit (Illumina, San Diego, CA) has been used for library preparation following the manufacturer’s instructions (library type: fr-firstrand). Final libraries were checked with both Qubit 2.0 Fluorometer (Invitrogen, Carlsbad, CA) and Agilent Bioanalyzer DNA assay. Libraries were then prepared for sequencing and sequenced on paired-end 150 bp mode on NovaSeq6000 (Illumina, San Diego, CA).

Base calling and demultiplexing was performed by Bcl2Fastq 2.20 version of the Illumina pipeline (<https://support.illumina.com/content/dam/illumina-support/documents/documentation/software_documentation/bcl2fastq/bcl2fastq2-v2-20-software-guide-15051736-03.pdf>). Adapter sequences were masked with Cutadapt v1.11 from raw fastq data, using the following parameters: anywhere (on both adapter sequences), overlap 5, times 2, minimum-length 35, mask-adapter (Martin, 2011). Illumina reads were trimmed, and bad quality samples were removed using BBDuk (<https://jgi.doe.gov/data-and-tools/bbtools/bb-tools-user-guide/bbduk-guide/>) by setting a minimum length of 35 bp and a minimum Phred-quality score of 25. Quality of the reads was checked before and after the trimming step using FastQC (<https://www.bioinformatics.babraham.ac.uk/projects/fastqc/>). The high quality reads were aligned against the *Oryza sativa Japonica* Group (IRGSP-1.0/Ensembl release 42) and *Oryza sativa Indica Group* (ASM465v1/Ensembl release 42) with STAR aligner (version 2.5.2b) (Dobin *et al.*, 2013). Therefore, each sample was mapped against each reference genome, obtaining two different BAM file groups.

Gene expression quantification was performed with featureCounts (version 1.5.1) (Liao et al., 2014). Normalization was applied to the raw fragment counts by using the Trimmed Mean of M -values (TMM) normalization and Fragments Per Kilobase Million (FPKM) normalization. Differential gene expression analysis was performed with edgeR (Robinson *et al.*, 2009). To identify differentially expressed genes (DEGs) a log2 fold change (FC) -0.5 ≤ or ≥ 0.5 was applied. Gene Ontology (GO) enrichment analysis was performed using AgriGO (Tian *et al.*, 2017) based on the hypergeometric statistical test, and a minimum significance of 0.05. Multiple testing corrections controlling false positives were also performed with the Hochberg (FDR) method. Enriched GO terms were clustered and plotted with the online analysis tool ReviGO (<http://revigo.irb.hr/>) (Supek e*t al.,* 2011).

*Identification of introgressed indica regions*

The identification of introgressed genomic regions was performed using an in-house bioinformatics pipeline at Sequentia Biotech (<http://www.sequentiabiotech.com>) to identify homozygous transcript variants. In the first step, transcript variants from the BAM files obtained from the alignment to *Oryza sativa Japonica* Group (IRGSP-1.0/Ensembl release 42) were detected. The BAM files for each genome were merged using samtools (<http://www.htslib.org/>) and grouped as OLESA and IL22. The MD tag was generated with SAMTOOLS as well. oPOSSUM (Sui Ho *et al.,* 2007) and Platypus (Rimmer *et al.*, 2014) were used to perform the Variant Calling Analysis from each merged BAM file obtained in the previous step. The parameters used in oPOSSUM were the following: ProperlyPaired False and SoftClipsExist True. The parameters used in Platypus were: filterDuplicates 0, minMapQual 0, minFlank 0, maxReadLength 500, minGoodQualBases 10, and minBaseQual 20. Then, all the variants in each gene were counted using Bedtools (Quinlan and Hall, 2010), intersectBed and FeatureCounts. The number of transcript variants in each gene in OLESA (indicating the natural variation of OLESA with respect to the *japonica* reference genome), and IL22 (representing both the *japonica* natural variation in OLESA and variations from *indica* introgressions) were identified. The RStudio software (<https://rstudio.com/>) was used to identify the *indica* introgressed genomic regions of IL22 by comparing the number of transcript variants in the IL22 and OLESA genotypes in each gene (grouped by orthologous genes between genomes). Those genes showing 10 or more transcript variants in IL22 than in OLESA were considered as *indica* introgressed genes, while those showing less than 10 transcripts variants were considered as *japonica* genes. The orthologous genes between the two reference genomes were obtained from Plant Ensembl BioMart.

**References**

Dobin, A., Davis, C. A., Schlesinger, F., Drenkow, J., Zaleski, C., Jha, S., et al. (2013). STAR: Ultrafast universal RNA-seq aligner. *Bioinformatics*. 29, 15-21. doi:10.1093/bioinformatics/bts635.

Liao, Y., Smyth, G. K., and Shi, W. (2014). FeatureCounts: An efficient general purpose program for assigning sequence reads to genomic features. *Bioinformatics*. 30, 923-930. doi:10.1093/bioinformatics/btt656.

Martin, M. (2011). Cutadapt removes adapter sequences from high-throughput sequencing reads. *EMBnet.journal*. 17, 10. doi:10.14806/ej.17.1.200.

Quinlan, A. R., and Hall, I. M. (2010). BEDTools: A flexible suite of utilities for comparing genomic features. *Bioinformatics*. 26, 841-842. doi:10.1093/bioinformatics/btq033.

Rimmer, A., Phan, H., Mathieson, I., Iqbal, Z., Twigg, S. R. F., Wilkie, A. O. M., et al. (2014). Integrating mapping-, assembly- and haplotype-based approaches for calling variants in clinical sequencing applications. *Nat. Genet.* 46, 912-918. doi:10.1038/ng.3036.

Robinson, M. D., McCarthy, D. J., and Smyth, G. K. (2009). edgeR: A Bioconductor package for differential expression analysis of digital gene expression data. *Bioinformatics*. 26, 139-140. doi:10.1093/bioinformatics/btp616.

Sui Ho, S. J., Fulton, D. L., Arenillas, D. J., Kwon, A. T., and Wasserman, W. W. (2007). OPOSSUM: Integrated tools for analysis of regulatory motif over-representation. *Nucleic Acids Res.* 35, 245-252. doi:10.1093/nar/gkm427.

Supek, F., Bošnjak, M., Škunca, N., and Šmuc, T. (2011). REVIGO Summarizes and Visualizes Long Lists of Gene Ontology Terms. *PLoS One.* 6, e21800. doi:10.1371/journal.pone.0021800.

Tian, T., Liu, Y., Yan, H., You, Q., Yi, X., Du, Z., et al. (2017). AgriGO v2.0: A GO analysis toolkit for the agricultural community, 2017 update. *Nucleic Acids Res.* 45, 122-129. doi:10.1093/nar/gkx382.
